# Surface polarization profile of ferroelectric thin films probed by X-ray standing waves and photoelectron spectroscopy

**DOI:** 10.1038/s41598-024-72805-1

**Published:** 2024-10-16

**Authors:** Le Phuong Hoang, Irena Spasojevic, Tien-Lin Lee, David Pesquera, Kai Rossnagel, Jörg Zegenhagen, Gustau Catalan, Ivan A. Vartanyants, Andreas Scherz, Giuseppe Mercurio

**Affiliations:** 1 European XFEL, 22869 Schenefeld, Germany; 2 Max Planck Institute for the Structure and Dynamics of Matter, 22761 Hamburg, Germany; 3 Institute of Experimental and Applied Physics, Kiel University, 24098 Kiel, Germany; 4 Department de Física, Universitat Autònoma de Barcelona, 08193 Bellaterra, Spain; 5 Diamond Light Source Ltd., Didcot, OX110DE Oxfordshire UK; 6 Catalan Institute of Nanoscience and Nanotechnology (ICN2), CSIC and BIST, Campus UAB, 08193 Bellaterra, Barcelona Spain; 7 Ruprecht Haensel Laboratory, Deutsches Elektronen-Synchrotron DESY, 22607 Hamburg, Germany; 8 Institucio Catalana de Recerca i Estudis Avançats (ICREA), 08010 Barcelona, Catalonia Spain; 9 Deutsches Elektronen-Synchrotron DESY, 22607 Hamburg, Germany

**Keywords:** Ferroelectric polarization, X-ray standing wave, X-ray photoelectron spectroscopy, Ferroelectrics and multiferroics, Surfaces, interfaces and thin films, Characterization and analytical techniques

## Abstract

Understanding the mechanisms underlying a stable polarization at the surface of ferroelectric thin films is of particular importance both from a fundamental point of view and to achieve control of the surface polarization itself. In this study, we demonstrate that the X-ray standing wave technique allows the surface polarization profile of a ferroelectric thin film, as opposed to the average film polarity, to be probed directly. The X-ray standing wave technique provides the average Ti and Ba atomic positions, along the out-of-plane direction, near the surface of three differently strained $\mathrm {BaTiO_3}$ thin films. This technique gives direct access to the local ferroelectric polarization at and below the surface. By employing X-ray photoelectron spectroscopy, a detailed overview of the oxygen-containing species adsorbed on the surface is obtained. The different amplitude and orientation of the local ferroelectric polarizations are associated with surface charges attributed to different type, amount and spatial distribution of the oxygen-containing adsorbates.

## Introduction

Ferroelectric thin films have attracted great scientific interest due to their properties, such as switchable polarization, ferroelasticity, piezoelectricity, and pyroelectricity, which are crucial for technological applications^1–4^. Displacive ferroelectrics, such as $\hbox {BaTiO}_3$ (BTO), exhibit an intrinsic spontaneous polarization associated with the relative displacement of cations and anions within the unit cell^5^. This polarization can be manipulated by varying the lattice parameters of the ferroelectrics^6,7^. To this end, ferroelectric thin films have been grown on substrates with different lattice constants. The lattice mismatch can induce uniform strain or strain gradients in the thin films^8^. Suitable substrates and bottom electrodes have been employed to tune the ferroelectric polarization, which is typically measured by piezoresponse force microscopy (PFM), a technique that is sensitive to the average polarization of the entire film, for thicknesses of a few tens of nanometers^9,10^. The distribution of the ferroelectric polarization at the surface, which can differ from that of the bulk, has been investigated theoretically as a function of various parameters, such as surface termination and adsorbates^11,12^. However, an experimental method that can simultaneously probe the surface ferroelectric polarization and the chemical composition of the adsorbates is still lacking.

The determination of the surface polarization has a twofold relevance. First, from a fundamental point of view, uncompensated charges at the surface of a ferroelectric thin film can be screened, among several mechanisms, by external charges provided by adsorbates^13–17^ or can lead to a reconstruction of the top unit cells to minimize the surface energy^18–20^. This, in turn, can influence the polarization of deeper layers, and, for very thin films, affect the polarization of the entire sample^21^. Therefore, in order to control the ferroelectric polarization of a thin film, probing it and understanding its stabilization mechanisms near the surface are of particular importance. Second, from the point of view of promising applications, ferroelectrics have been proposed as catalysts with chemical activity that is switchable between reducing and oxidizing surfaces depending on the polarization direction^22–27^. In this context, determining the surface polarization is the first step towards the development of efficient ferroelectric catalysts.

Among non-destructive techniques employed to determine atomic positions at (and near) the surface, and thus the microscopic origin of ferroelectric polarization, crystal truncation rod (CTR) scattering and low-energy electron diffraction (LEED-IV) have been successfully employed to reveal atomic structures with approximately $\pm 10\hbox { pm}$ accuracy^13–15,18,28^. However, neither of the above methods provides spectroscopic information on atoms in ferroelectric thin films and adsorbate species. This could be achieved by X-ray photoelectron diffraction, however, at the expense of rather complex multiple-scattering simulations^29–33^.

In this work, we employ the X-ray standing wave (XSW) technique, a combination of X-ray diffraction (XRD) and X-ray spectroscopy, to determine atomic positions with picometer accuracy and chemical specificity. The structural accuracy of this technique for determining atomic positions in single crystals and adsorbates on crystal surfaces has been demonstrated extensively^34–38^. Furthermore, the XSW technique proved to be successful in determining the average polarity of non-centrosymmetric single crystals^39^ and thin films^40,41^, as well as ferroelectric thin films^42,43^. In the latter experiments, the average polarity of the thin films was determined by combining XSW and X-ray fluorescence spectroscopy (XFS). Here, we apply the XSW technique, in combination with X-ray photoelectron spectroscopy (XPS), a more surface sensitive technique than XFS, to measure the displacement of Ti atoms from the center of the unit cell in differently strained BTO thin films, and thereby deduce the ferroelectric polarization at different depths near the surface. These data are interpreted in the context of the average film polarization measured by PFM, as well as the type, content and spatial distribution of adsorbates on the sample surface. This combination of the structural sensitivity of XSW with the chemical specificity and depth selectivity of XPS, provides a detailed insight into the near-surface polarization profile and its interplay with adsorbates and the bulk ferroelectric polarization.

## X-ray standing waves generated in thin films

**Figure 1:**
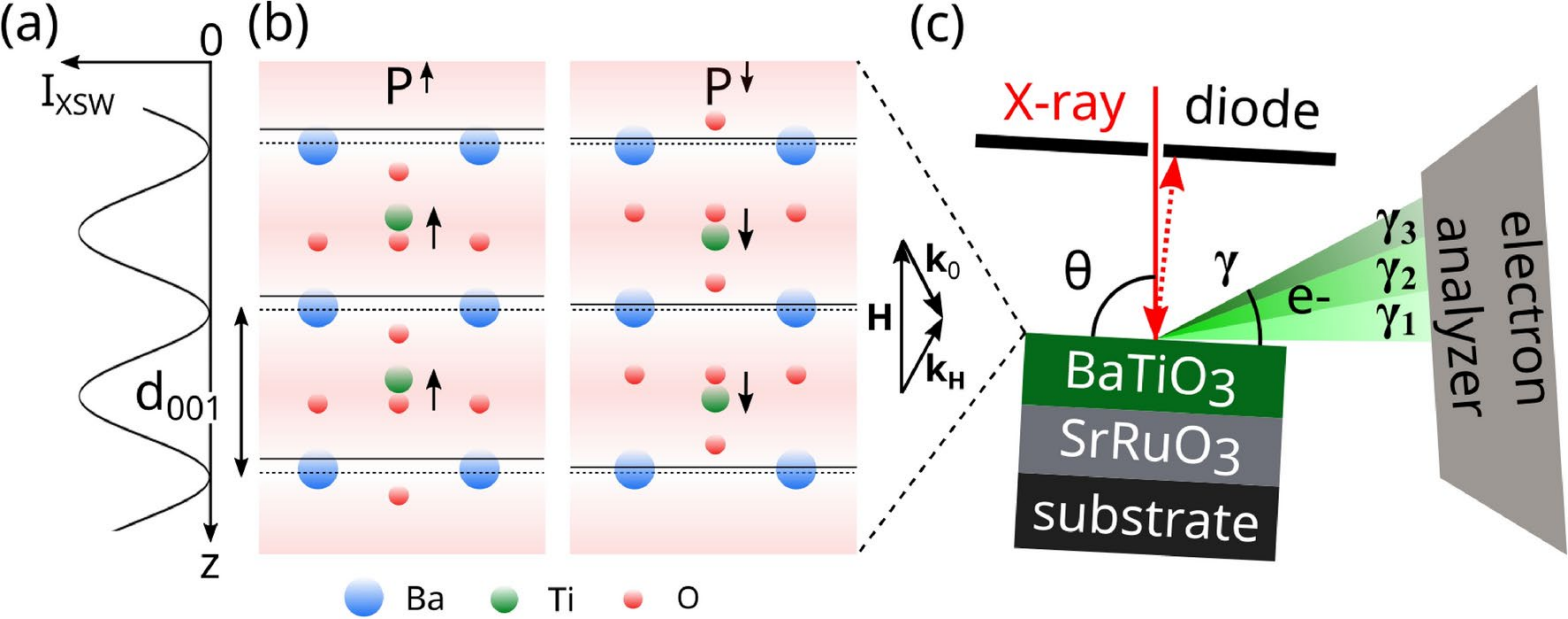
(**a**) XSW intensity of BTO (001) Bragg reflection and *z* axis orientation, with $z = 0$ at the sample surface. (**b**) Side view of the top two BTO unit cells with ferroelectric polarization $\textrm{P}^\uparrow$ and $\textrm{P}^\downarrow$, and Bragg spacing $d_{001}$. In the $\textrm{P}^{\uparrow }$ sketch, Ti and equatorial O atoms are offset by 0.05*c*, above and below the center of the unit cell, respectively, while apical O atoms are offset by $-0.1c$. The same offsets in the opposite direction apply to the $\textrm{P}^{\downarrow }$ sketch. Dashed lines indicate Ba planes, while solid lines refer to the Bragg diffraction planes and their absolute position with respect to the Ba plane is given by $c(\beta _h + \varphi _0)/(2\pi )$ (Section Ba and Ti XSW). Here, $\beta _h^{\uparrow }/(2\pi ) = 0.17$, $\beta _h^{\downarrow }/(2\pi ) = 0.11$, and $\varphi _0/(2\pi ) = 0.08$, which is the average of $\varphi _0/(2\pi )$ found in the three samples (“Methods”, “Deformation phase calculation”) and is taken as an example. (**c**) Sketch of the experimental setup (top view) used at the beamline I09 of the Diamond Light Source, including sample, electron analyzer, and photodiode. The photodiode was located 10 mm away from the sample and was equipped with an Al mask in front to minimize the fluorescence background. The Bragg angle $\theta$ and the photoelectron exit angle $\gamma$ are shown, together with photoelectron exit angle ranges $\gamma _1$, $\gamma _2$ and $\gamma _3$, incident $\varvec{k}_0$ and Bragg-diffracted $\varvec{k}_{\varvec{H}} = \varvec{k}_0 + \varvec{H}$ X-ray wavevectors.

The XSW technique is particularly useful for determining atomic positions in crystals, surfaces, and their adsorbates^34–38^. The interference between incoming and Bragg-diffracted X-ray plane waves in a perfect crystal results in an X-ray standing wave field with the following sinusoidal modulation of the X-ray intensity $I_\textrm{XSW}$ (Fig. 1a,b):

1 $$\begin{aligned} I_\textrm{XSW}\left( E_\nu \right) \propto 1 + \left| \frac{\mathscr {E}_\textrm{H}}{\mathscr {E}_\textrm{0}}\right| ^2 + 2 \left| \frac{\mathscr {E}_\textrm{H}}{\mathscr {E}_\textrm{0}}\right| \cos \left( \alpha (E_\nu ) + \varvec{h}\varvec{r} \right) , \end{aligned}$$

where $\varvec{h} = 2 \pi \varvec{H}$, and $\varvec{H}$ is the reciprocal lattice vector. In Eq. (1), the three terms represent the incident, Bragg-diffracted, and interference X-ray wave, respectively. As the incident photon energy $E_\nu$ varies through the (hkl) Bragg reflection, the phase $\alpha (E_\nu )$ between the Bragg-diffracted $\mathscr {E}_\textrm{H}$ and incident $\mathscr {E}_0$ electric field amplitudes changes by $\pi$. This leads to a shift of the XSW field along $\varvec{H}$ by $d_\textrm{hkl}/2$, where $d_\textrm{hkl} = |\varvec{H} |^{-1}$ is the spacing between two consecutive (hkl) atomic planes (Fig. 1b). Atoms at different positions in the unit cell experience different X-ray absorption and hence give rise to different photoelectron (PE) yield as a function of the photon energy $E_\nu$. As a result, the atomic positions can be determined with picometer spatial resolution by monitoring the corresponding PE yield (see “Results”).

In a typical ferroelectric thin film grown on a substrate, the lattice mismatch may lead to strain gradients in the epitaxial layers^8^. Therefore, thin films are generally characterized by a deformation field $\varvec{u}\left( z\right)$, which defines the actual displacement of atoms from the corresponding position in a perfect crystal, and the static Debye–Waller factor $\textrm{e}^{-W(z)}$, which accounts for random displacement of atoms from their average position along the *z* direction (Fig. 1a). In contrast to the perfect crystal case above, the XSW generated in a thin film is modified by the deformation phase $\varphi \left( z\right) = \varvec{h} \cdot \varvec{u}\left( z\right)$ due to the crystal deformation field. Based on the dynamical theory of diffraction, the Takagi–Taupin equations^44–46^ describe the propagation of X-rays in a deformed crystal, and thus give the following XSW intensity generated in a typical ferroelectric thin film:

2 $$\begin{aligned} I_\textrm{XSW}\left( E_\nu ,z\right) = 1 + R\left( E_\nu ,z\right) + 2C \sqrt{R\left( E_\nu ,z\right) } \textrm{e}^{-W\left( z\right) }\cos \left( \alpha (E_\nu ,z) + \varphi \left( z\right) + 2\pi z/d_\textrm{hkl} \right) , \end{aligned}$$

where $R_0(E_\nu ) = R(E_\nu ,0)$ indicates the observable X-ray diffracted intensity at the sample surface ($z=0$), and the parameter *C* depends on the X-ray polarization^47^ (see “Methods”, “Reflection and transmission calculation”). Based on the XSW generated in a ferroelectric thin film of thickness* t*, from the PE yield curve

3 $$\begin{aligned} \begin{aligned} \kappa ^s_\gamma (E_\nu ) = I_0^{-1} \int _{0}^{t_{L_0}} dz \rho _\textrm{yi}\left( E_\nu ,z,\gamma \right) |T\left( E_\nu ,z\right) |^2 \Big [1 + R\left( E_\nu ,z\right) + 2C \sqrt{R\left( E_\nu ,z\right) } F^s_{c,\gamma } \cos \left( \alpha (E_\nu ,z) + \varphi _0 + 2 \pi P^s_{c,\gamma } \right) \Big ], \end{aligned} \end{aligned}$$

we can determine the average position and distribution of atoms *s*, which are defined as the coherent position $P^s_{c,\gamma }$ and coherent fraction $F^s_{c,\gamma }$, respectively. These parameters are equivalent to the phase and amplitude of the structure factor $\varvec{S}^s_{\varvec{h}} = \sum _{j} \exp (\textrm{i}\varvec{h} \varvec{r}_j^s ) = \left| \varvec{S}^s_{\varvec{h}}\right| \exp (\textrm{i} \varphi ^s_{\varvec{h},\gamma })$ referring to the positions $\varvec{r}_j^s$ of atoms *s* in the unit cell. More specifically, $P^s_{c,\gamma } = \varphi ^s_{\varvec{h},\gamma } / (2 \pi )$ and $F^s_{c,\gamma } = |\varvec{S}^s_{\varvec{h}}| {\textrm{e}^{-W_0} \textrm{e}^{-W^\textrm{T}}}$, where $\textrm{e}^{-W_0}$ and $\textrm{e}^{-W^\textrm{T}}$ are the Debye–Waller factors accounting for static and thermal atomic displacements. Therefore, the absolute average position of atoms *s* within the unit cell along $\varvec{H}$ is given by $z^s_\gamma = P^s_{c,\gamma } d_\textrm{hkl}$, and their spatial distribution is characterized by $F^s_{c,\gamma }$, with $0<P^s_{c,\gamma }<1$ and $0<F^s_{c,\gamma }<1$. In particular, $F^s_{c,\gamma } = 1$ refers to all atoms at the same *z*, while $F^s_{c,\gamma } = 0$ corresponds to a uniform distribution of two or more atomic positions across the unit cell.

In Eq. (3), the PE yield function $\kappa ^s_\gamma (E_\nu )$ is the sum of yield contributions from atoms in the top layer $L_0$ at positions $0<z<t_{L_0}$, weighted by $\rho _\textrm{yi}(E_\nu ,z,\gamma )$, with XSW transmission $T\left( E_\nu ,z\right)$, and normalization factor $I_0 = \int _{0}^{t_{L_0}} dz \rho _\textrm{yi}(E_\nu ,z,\gamma )$. The function $\rho _\textrm{yi}(E_\nu ,z,\gamma ) = \exp \left( -z/\lambda _{l,\gamma }\right)$ gives the probability of detecting a photoelectron from the atomic core level *l* at depth *z*, with exit angle $\gamma$ from the sample surface (Fig. 1c). The parameter $\lambda _{l,\gamma } = \lambda _{l}(E_\nu ) \sin \gamma$ is the electron escape depth^48^, while $\lambda _{l}\left( E_\nu \right)$ indicates the inelastic mean free path (IMFP), or more correctly the effective attenuation length (EAL) that includes elastic scattering effects^49^. Tuning $\gamma$ allows the surface sensitivity to be varied and provides average atomic positions over different depths from the surface. To determine the average distribution of atoms *s* at a given exit angle $\gamma$, the measured PE yield curve is fitted with Eq. (3) using $P^s_{c,\gamma }$ and $F^s_{c,\gamma }$ as fit parameters. In fact, all other quantities in Eq. (3) can be calculated from known sample properties (see “Methods”, “Reflection and transmission calculation”), or derived from the fit of Bragg reflectivity data, e.g., the average amorphization $\textrm{e}^{-W_0}$ of layer $L_0$ and the corresponding deformation phase $\varphi _0$ (see “Results” and “Methods”, “Deformation phase calculation”).

## Results

### Sample characterization

**Figure 2:**
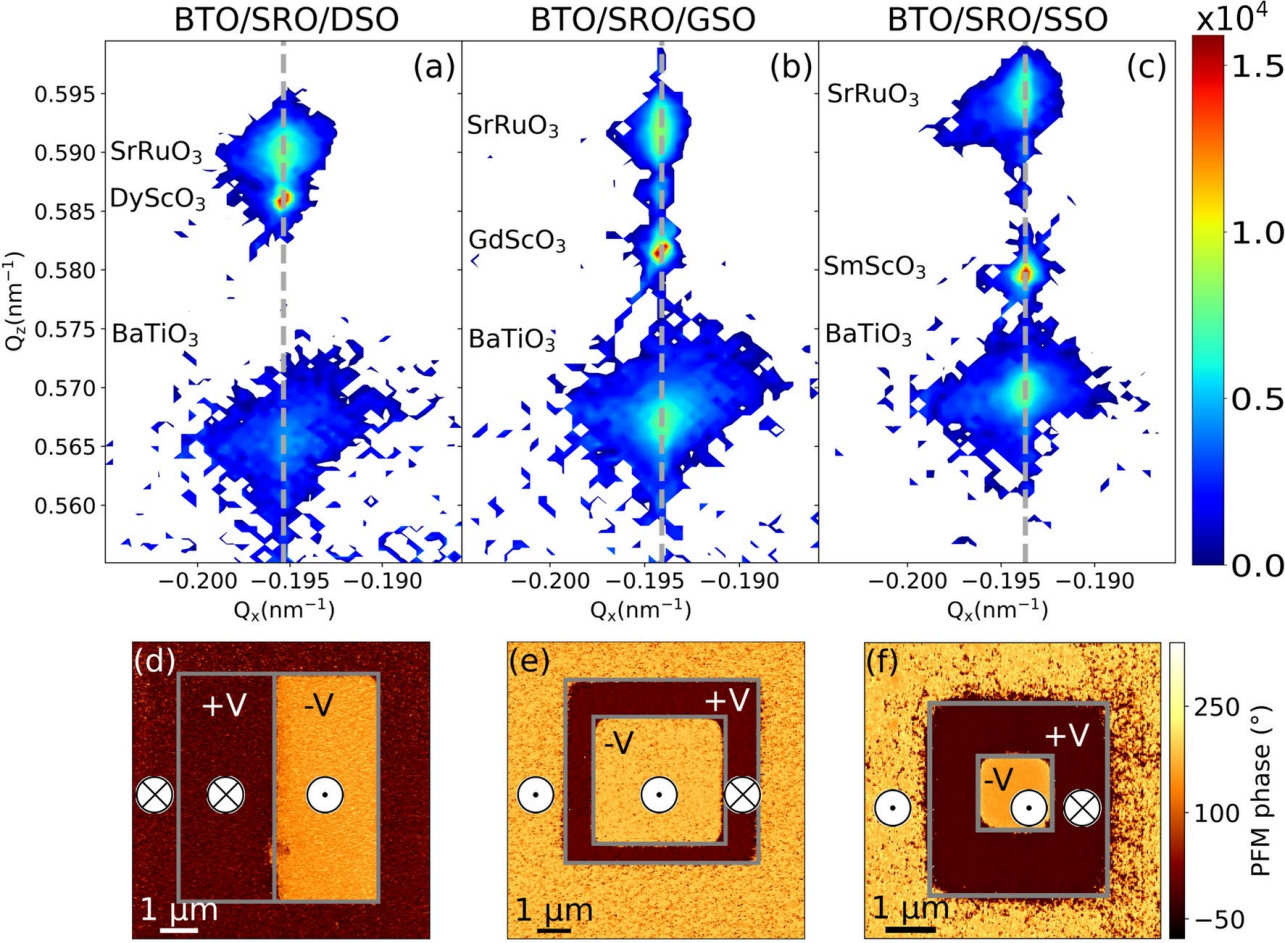
Reciprocal space maps around (-103) substrate Bragg peak of the BTO/SRO/DSO (**a**), BTO/SRO/GSO (**b**) and BTO/SRO/SSO (**c**) samples. The gray vertical dashed lines indicate the reciprocal lattice parameter $Q_\textrm{x}$ shared by the substrate, the BTO, and SRO thin films in each sample. PFM phase images of the BTO/SRO/DSO (**d**), BTO/SRO/GSO (**e**), and BTO/SRO/SSO (**f**) samples. The sign of the applied tip voltage ($\pm \textrm{V}$) within the gray boxes and the resulting average polarization direction $\textrm{P}^\uparrow$ ($\odot$) or $\textrm{P}^\downarrow$ ($\otimes$) in the probed areas are marked on each panel. The PFM phase beyond the gray boxes indicates the average polarization direction of as-grown samples.

The samples investigated in this work are BTO ferroelectric thin films grown on three different substrates $\hbox {DyScO}_3$ (DSO), $\hbox {GdScO}_3$ (GSO), and $\hbox {SmScO}_3$ (SSO), with a $\hbox {SrRuO}_3$ (SRO) thin film in between serving as the bottom electrode. Sample growth was performed by means of pulsed laser deposition (see “Methods”, “Sample growth”) on substrates with (001) orientation according to the pseudocubic notation^50^. At the given growth conditions, BTO thin films have mixed (BaO and $\hbox {TiO}_2$) termination. The thickness of the BTO and SRO thin films was determined by grazing X-ray reflectivity (see “Methods”, “Grazing X-ray reflectivity” and Supplementary Fig. 1). The SRO layers have thicknesses in the range of 20–26 nm, while the BTO layers are 20 nm, 37 nm, and 35.5 nm thick on DSO, GSO, and SSO, respectively (Table 1). To determine the average in-plane strain of the thin films, X-ray reciprocal space maps (RSM) around the (-103) substrate Bragg peak were measured (see “Methods”, “X-ray reciprocal lattice map”). Results reported in Fig. 2a–c show that all BTO and SRO thin films are coherently strained to their underlying substrates, without any relaxation of the in-plane lattice parameter. The in-plane strain applied by a substrate to the BTO thin film is calculated as $\varepsilon ^{a}_\textrm{BTO} = \left( a_\textrm{BTO} - a_\textrm{b,BTO} \right) / a_\textrm{b,BTO}$, by comparing the measured in-plane lattice parameter of the thin film $a_\textrm{BTO}$ (see “Methods”, “X-ray reciprocal lattice map”) with the respective bulk value $a_{b,\textrm{BTO}} = 3.992$ Å^5^. As a result, the in-plane compressive strain in the BTO thin film is smallest on the SSO substrate ($-0.38\%$, $a_{b,\textrm{SSO}} =$ 3.977 Å^51^), it increases on GSO ($-0.63\%$, $a_{b,\textrm{GSO}} =$ 3.967 Å^51^), and is largest on DSO ($-1.23\%$, $a_{b,\textrm{DSO}} =$ 3.943 Å^51^) (Table 1). The in-plane compressive strain in BTO thin films induces an out-of-plane spontaneous ferroelectric polarization^6^. To determine the average polarization orientation of the as-grown BTO thin films, and to verify that all samples can be electrically switched, piezoresponse force microscopy (PFM) was employed. PFM data (Fig. 2d–f and “Methods”, “Piezoresponse force microscopy”) show that the average BTO polarization of BTO/SRO/DSO is down ($\textrm{P}^\downarrow$), while in the other two samples it is up ($\textrm{P}^\uparrow$), i.e., with the Ti atom below ($\textrm{P}^\downarrow$) or above ($\textrm{P}^\uparrow$) the center of the oxygen octahedra (Fig. 1b). The different average polarization direction of the three BTO samples results from the complex interplay of the electronic structure and the chemistry of the surface and the interface to the bottom layer^11,52,53^. Furthermore, both the absence of side peaks in RSM data and the presence of a homogeneous as-grown phase measured by PFM support the presence of a single domain in our samples. This ensures that atomic positions of the same structural phase are measured by XSW measurements. Finally, the analysis of AFM images provides a root mean square surface roughness $S_\textrm{RMS}$ of approximately 0.84(8) nm (Table 1), in line with Ref.^54^.

**Table 1:** BTO and SRO layer thicknesses, $t_\textrm{BTO}$ and $t_\textrm{SRO}$, resulting from grazing X-ray reflectivity (see “Methods”, “Grazing X-ray reflectivity” and Supplementary Fig. 1). All substrates are 0.5 mm thick. The root mean square surface roughness $S_\textrm{RMS}$ of BTO thin films was determined from AFM images of $3.9\times 3.9\,\upmu \hbox {m}^2$ area. Compressive in-plane strain in BTO ($\varepsilon ^{a}_\textrm{BTO}$) thin films, measured by X-ray reciprocal space maps (Fig. 2a–c). Orientation of the average BTO polarization $\textrm{P}$ measured by PFM (Fig. 2d–f).

Acronym	Sample	$t_\textrm{BTO}$ (nm)	$S_\textrm{RMS}$ (nm)	$t_\textrm{SRO}$ (nm)	$\varepsilon ^{a}_\textrm{BTO}$ (%)	$\textrm{P}$
BTO/SRO/DSO	$\hbox {BaTiO}_3$/$\hbox {SrRuO}_3$/$\hbox {DyScO}_3$	20	0.95	26	-1.23	$\downarrow$
BTO/SRO/GSO	$\hbox {BaTiO}_3$/$\hbox {SrRuO}_3$/$\hbox {GdScO}_3$	37	0.77	24	-0.63	$\uparrow$
BTO/SRO/SSO	$\hbox {BaTiO}_3$/$\hbox {SrRuO}_3$/$\hbox {SmScO}_3$	35.5	0.81	20	-0.38	$\uparrow$

### X-ray standing wave setup

XSW experiments were performed at the I09 beamline of Diamond Light Source^55^. The soft X-ray branch of I09, equipped with a plane grating monochromator, delivered an X-ray beam of approximately $300\,\upmu \hbox {m} \times 200\,\upmu \hbox {m}$ full width at half maximum (FWHM) at the sample. Each XSW measurement consisted of recording Bragg reflections and simultaneously photoelectron spectra of the sample. The Bragg reflections were measured by scanning the photon energy $E_\nu$ with the incoming X-ray beam impinging on the sample at a fixed angle of incidence ${\theta } = 87^{\circ }$ (Fig. 1c). The intensity of the diffracted X-ray beam was measured by a Si photodiode with a central through hole for the incident beam to pass (Fig. 1c). With this experimental geometry, the (001) Bragg reflections of the BTO and SRO films and the substrates were recorded within the range of photon energy $E_\nu$ from 1400 eV to 1700 eV. Simultaneously, XPS spectra were measured by a Scienta EW4000 electron analyzer, with the detection system consisting of a microchannel plate (MCP) followed by a charge-coupled device (CCD). The wide acceptance angle of the electron analyzer enabled parallel measurements of spectra over three different exit angle ranges: $\gamma _1$ ($7.8^{\circ } \pm 5.4^{\circ }$), $\gamma _2$ ($18.5^{\circ } \pm 5.4^{\circ }$) and $\gamma _3$ ($27.4^{\circ } \pm 3.6^{\circ }$). This provides the chemical and structural information of the BTO films with increasing depth sensitivity from $\gamma _1$ to $\gamma _3$ (Fig. 1c). The overall spectral energy resolution, limited by the X-ray bandwidth, was approximately 400 meV. The (001) Bragg reflections, XPS and XSW experimental results are reported in the following sections.

### (001) Bragg reflections

**Figure 3:**
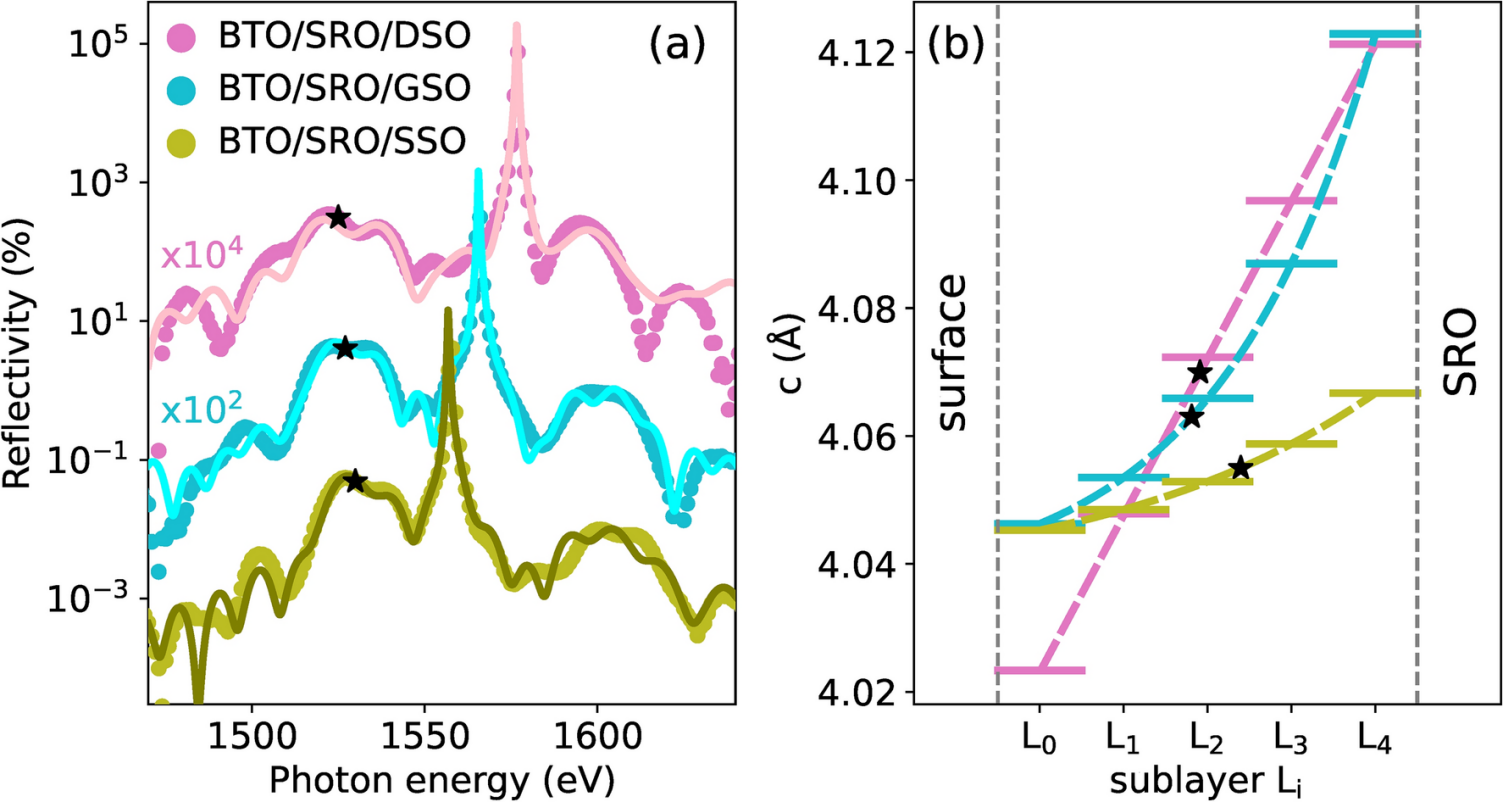
(**a**) (001) Bragg reflectivity $R_0(E_\nu )$ (points) and corresponding fit curves (solid lines) of samples BTO/SRO/DSO (pink), BTO/SRO/GSO (cyan) and BTO/SRO/SSO (green). (**b**) BTO out-of-plane lattice parameter $c_i$ (solid lines) in sublayers $L_i$ and *c*(*z*) (dotted lines) based on Eq. (4) and a linear distribution of *c* (see Section (001) Bragg reflections). The average BTO out-of-plane lattice parameters $\overline{c}_\textrm{BTO}$ are marked by black stars.

To determine the atomic positions at the BTO surface along the out-of-plane polarization direction, the (001) Bragg reflection was chosen for the XSW experiments. Figure 3a shows the reflectivity curves around the (001) reflections of the BTO, SRO, and substrates. The reflections of the substrates are more than two orders of magnitude stronger and much narrower compared to those of the thin layers. The low intensity and the broadening of the thin film Bragg peaks result from two factors: the finite film thickness and the inhomogeneous strain. More specifically, the reflectivity of BTO [SRO] films lies in the range of $0.03\% {-} 0.05\%$ $\left[ 0.01\% {-} 0.02\% \right],$ whereas the substrate reflectivity is of the order of $10\%$ for all samples. Going from SSO, to GSO, and then to DSO, the substrate Bragg peak gradually shifts to higher photon energies, as expected from the decreasing trend of the bulk out-of-plane lattice parameters of the substrates (Fig. 2a–c). This trend suggests that the in-plane strain in the films becomes more compressive in BTO and less tensile in SRO (see Supplementary Note 1) in the same substrate order. As a result, the average *c* parameters of BTO (see “Methods”, “[Sec Sec18]”) increase from the SSO to the DSO sample: $\overline{c}_\textrm{BTO} =$ 4.055 (22) Å, 4.063(23) Å, 4.070(45) Å, respectively. These values are in quantitative agreement with Ref.^6^ and with calculations, assuming a Poisson’s ratio of 0.31^56^. For the BTO thin films, the measured $\overline{c}_\textrm{BTO}$ parameters correspond to an average out-of-plane strain $\overline{\varepsilon }^c_\textrm{BTO} = \left( \overline{c}_\textrm{BTO} - c_{b,\textrm{BTO}} \right) / c_{b,\textrm{BTO}}$ of $0.48\%$, $0.68\%$ and $0.84\%$ for SSO, GSO and DSO, respectively, with the bulk out-of-plane lattice parameter $c_{b,\textrm{BTO}} =$ 4.036 Å^5^.

As anticipated above, an epitaxial thin film may be characterized by an inhomogeneous out-of-plane strain. According to the general strain profile model discussed in Refs.^8,57^, the strain gradient is expected to be proportional to the strain, ${\partial \varepsilon }^c/{\partial z} \propto \varepsilon ^c$, independently of the actual relaxation mechanism. As a result, the out-of-plane parameter *c* follows an exponential dependence on *z*:

4 $$\begin{aligned} c(z) = c_b \left( 1 + \varepsilon _\textrm{int}^c \textrm{e}^{-(t-z)/\delta } \right) , \end{aligned}$$

where $\varepsilon _\textrm{int}^c = (c_\textrm{int} -c_b)/c_b$ is the strain at the interface with the underlying layer (or substrate), and $\delta$ is the penetration depth of strain that is inversely proportional to the strain gradient. This model has been successfully applied to ferroelectric thin films of several 100s nm^8,57^. In a recent work^58,59^, it was also found that 50 nm thick $\mathrm {PbTiO_3}$ films, displaying high crystalline quality and no indication of in-plane relaxation, could be well described by an exponential profile of *c* parameters, as further confirmed by TEM images. In this case^59^, the strain gradient was assigned to a compositional gradient of lead oxide dipolar vacancies. A similar distribution of vacancies or defects could be present also in our samples and may underlay the presence of strain gradients. In fact, modelling our BTO and SRO thin films with a constant $\overline{c}$ leads to unsatisfactory fit results. To fit the experimental reflectivity curves in Fig. 3, BTO and SRO layers are divided into *n* sublayers $L_i$ (with $i = 0, \ldots , n-1$) of equal thickness $t_i$ with an out-of-plane lattice parameter $c_i$ varying exponentially with *i* as described by Eq. (4), Debye–Waller factor $\textrm{e}^{-W_i}$, and deformation phase $\varphi _i = 2 \pi ( c_i - \overline{c})t_i/\overline{c}^2$ (see “Methods”, “Deformation phase calculation”). In our samples, the minimum common number of sublayers necessary to accurately describe them is $n=5$. Increasing the number of sublayers *n* does not improve the fit. For the BTO thin film of the BTO/SRO/DSO sample, both the exponential *c* distribution of Eq. (4) and the linear *c* distribution $\hbox {c(z)} = c_b \left( 1 + \varepsilon _\textrm{int}^c -\delta _\textrm{lin}(t-z) \right)$ were employed and compared in Supplementary Fig. 3. Here, $\delta _\textrm{lin}$ is the rate of strain change with *z*. We observe that a linear *c* distribution can reproduce the experimental data better than the exponential one for the $20\hbox { nm}$ BTO layer in BTO/SRO/DSO. A similar observation based on TEM studies was reported for films with thickness $\le 20\hbox { nm}$^60,61^. We note also that no improvement in the fit was observed when applying the linear distribution to the thicker BTO layers or to the bottom SRO layers. Experimental data in Fig. 3a are fitted with the reflectivity $R_0(E_\nu )$ using the fitting parameters $\textrm{e}^{-W_i}$ ($i=0, \ldots ,4$), $\varepsilon _\textrm{int}^c$ and $\delta$ (or $\delta _\textrm{lin}$) for the BTO and SRO layer (see Section X-ray standing waves generated in thin films and “Methods”, “Reflection and transmission calculation”).

The best fits to the reflectivity curves shown in Fig. 3a reproduce reasonably well the main features of the experimental data, with deviations $$< 5 \times 10^{-3}\%$$. This validates the models used to capture the main structural features of our samples. However, the small differences between experimental data and fit curves suggest a more complex strain distribution which cannot be mimicked by the relatively simple strain models presented above. The resulting out-of-plane parameters $$c_i$$ are shown in Fig. 3b and in Supplementary Fig. 4. The Debye–Waller factors $$\textrm{e}^{-W_i}$$ in BTO sublayers are mostly $$\ge 0.9$$ with lower values at the interface to SRO (Supplementary Table 1). A similar observation of larger structural disorder at the interface to the layer below has been revealed by transmission electron microscopy (TEM) studies^60–62^. The fit parameters $$\varepsilon _\textrm{int}^c$$ and $$\delta$$ of BTO, in the BTO/SRO/GSO and BTO/SRO/SSO samples, show a direct and inverse proportionality to the in-plane compressive strain $$\varepsilon ^{a}_\textrm{BTO}$$, respectively (Supplementary Table 2). In fact, a larger in-plane compressive strain $$\varepsilon ^a_\textrm{BTO}$$ leads to larger average out-of-plane strain $$\overline{\varepsilon }^c_\textrm{BTO}$$ and therefore, a larger strain gradient. Similar and even larger strain gradients, as compared to $${\partial \varepsilon ^c}/{\partial z} =$$$$1.5\times 10^{-4}\hbox { nm}^{-1}$$ in BTO/SRO/DSO, have been previously observed by TEM and CTR scattering measurements on BTO thin films^15,60^ and other ferroelectrics^59,61,62^. Interestingly, despite the different in-plane compressive strains in the three samples (Table 1), the strain gradients lead to similar out-of-plane lattice parameters $$c_0$$ at the top sublayer $$L_0$$: 4.02 Å, 4.046 Å, 4.045 Å. Subsequently, the fitting of the experimental reflectivity curves in Fig. 3a provides the necessary structural data to calculate the PE yield fit function $$\kappa ^s_\gamma (E_\nu )$$ in Eq. (3). We turn now to the determination of the experimental PE yield from XPS spectra.

### Ba, Ti and O XPS

**Figure 4:**
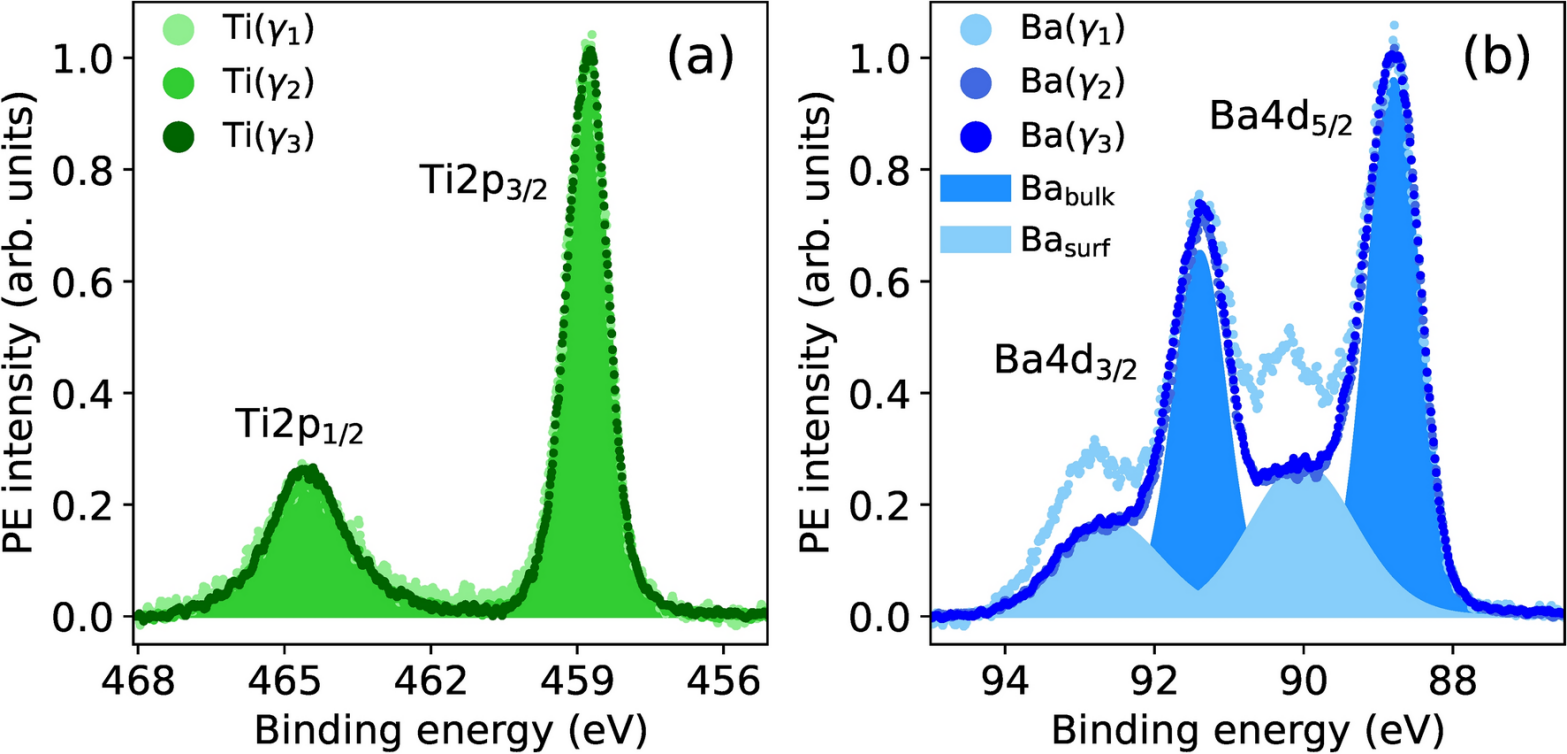
PE spectra of Ti 2p (**a**) and Ba 4d (**b**) core levels measured with $E_\nu = 1420\hbox { eV}$ at the exit angle ranges $\gamma _1$, $\gamma _2$, and $\gamma _3$, on the BTO/SRO/DSO sample. Each spectrum is normalized to the respective PE intensity maximum. Shaded component areas refer to spectra measured at the exit angle range $\gamma _3$. The $\mathrm {Ba_{surf}}$ [$\mathrm {Ba_{bulk}}$] component refers to Ba atoms at [below] the top BaO atomic plane. Similar PE spectra measured on the BTO/SRO/GSO and BTO/SRO/SSO samples are reported in Supplementary Fig. 5.

The photoelectron yield $\kappa ^s_\gamma (E_\nu )$ of atomic species *s*, measured at exit angle range $\gamma$ and photon energy $E_\nu$, is defined as the corresponding PE peak integral after background subtraction. To determine the PE yield of Ba and Ti atoms in the BTO thin films, Ba 4d and Ti 2p PE spectra were measured over the three exit angle ranges ($\gamma _1$, $\gamma _2$, $\gamma _3$), and the results are reported in Fig. 4. The Ti spectra in Fig. 4a show the 2p doublet at 458.8 and 464.5 eV. Spectra measured at different exit angles show the same spectral shape. This indicates that Ti atoms at the BTO surface and below experience the same chemical environment for the formation of the nominal $\hbox {Ti}^{4+}$ state. While this is expected for Ti atoms below the surface, it holds true also for surface Ti atoms bound to oxygen-containing adsorbates^14,63,64^. In addition, the absence of a peak at 1.7 eV below the main Ti 2$\hbox {p}_{3/2}$ peak provides evidence for the absence of oxygen vacancies leading to $\hbox {Ti}^{3+}$ near the BTO surface^14,63^. In contrast, Ba 4d spectra, displayed in Fig. 4b, show at least two kinds of atomic species. The spin-orbit split levels Ba 4$\hbox {d}_{5/2}$ and Ba 4$\hbox {d}_{3/2}$ at 88.8 eV and at 91.4 eV originate from the Ba atoms below the surface and are hence referred to as the bulk component ($\mathrm {Ba_{bulk}}$). On the other hand, the $\mathrm {Ba_{surf}}$ peaks exhibit a binding energy shift $\Delta \textrm{BE}=+$ 1.2 eV and twice the FWHM compared to the bulk component. The enhancement of $\mathrm {Ba_{surf}}$ in the most surface sensitive spectrum (Ba($\gamma _1$)) clearly indicates its correspondence to Ba atoms at the BTO surface, as observed in previous studies^65,66^. The larger FWHM is consistent with a continuity of chemical environments surrounding Ba atoms at the surface^66^, which could be related to different species such as $\hbox {OH}^-$, at different adsorption sites^11,12^, and $\mathrm {O_2}^-$ species^65^, as suggested by the correlation with oxygen components (see Supplementary Note 4). As a result, the PE yield with the largest contribution from the surface unit cell ($\gamma _1$) is given by the sum of the two components ($\mathrm {Ba_{surf}} + \mathrm {Ba_{bulk}}$) in order to include both Ba atoms at the surface and just below it, while the Ba PE yield of the deeper unit cells (at $\gamma _2$ and $\gamma _3$) is given only by the $\mathrm {Ba_{bulk}}$ component. On the other hand, the Ti PE yield is given by the total area of the Ti doublet for all exit angle ranges. As shown above, the possibility provided by XPS to distinguish between atoms in different chemical environments enables the XSW technique to selectively determine the positions of different chemical species of the same element. This is shown in more detail in Section Ba and Ti XSW.

**Figure 5:**
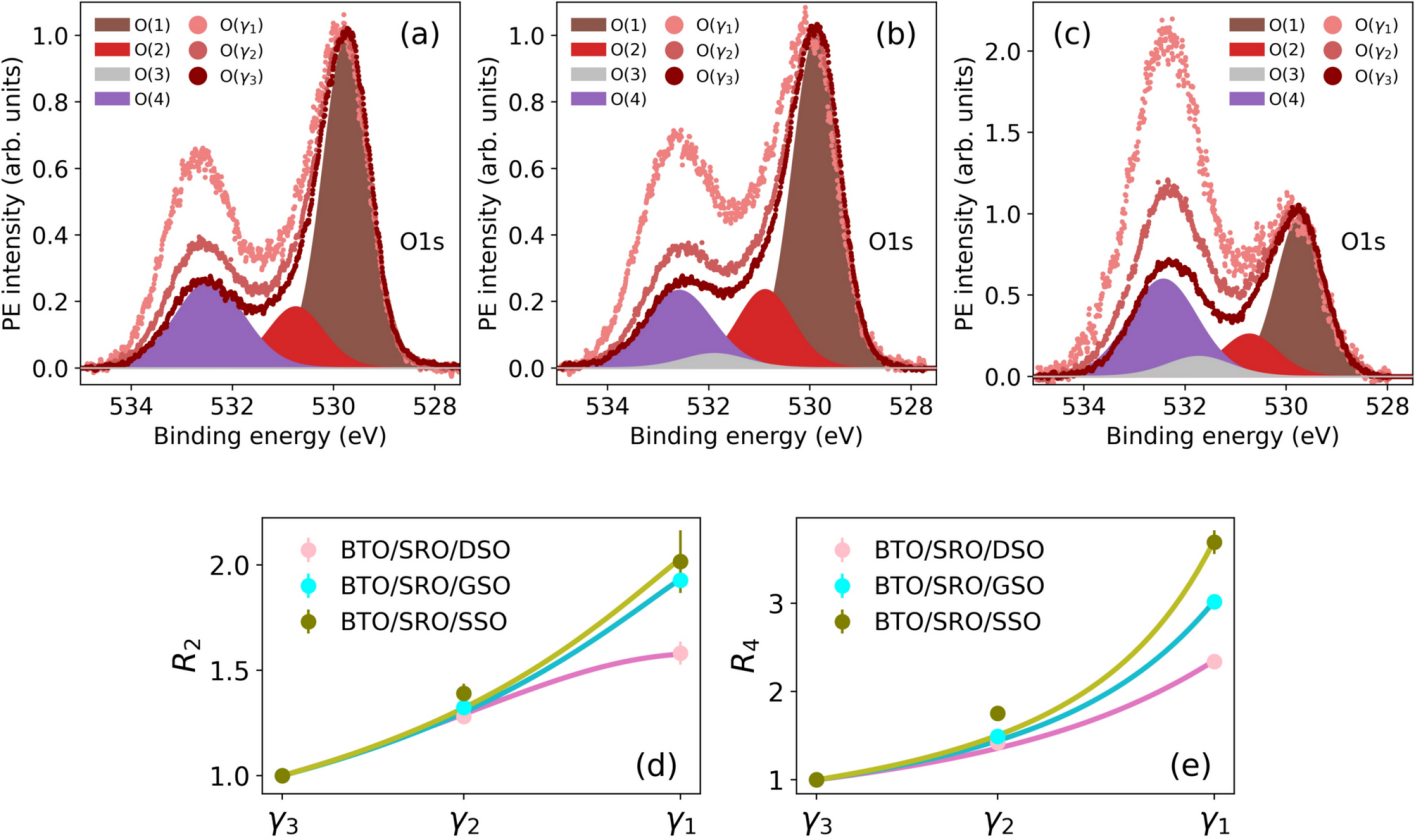
O 1s PE spectra $\textrm{O}(\gamma _1)$, $\textrm{O}(\gamma _2)$ and $\textrm{O}(\gamma _3)$ integrated over the respective exit angle ranges in the BTO/SRO/DSO (**a**), BTO/SRO/GSO (**b**), and BTO/SRO/SSO (**c**) samples. Each spectrum is normalized to the respective maximum PE intensity. Shaded component areas $\mathrm {O(1)}$, $\mathrm {O(2)}$, $\mathrm {O(3)}$, and $\mathrm {O(4)}$ refer to $\textrm{O}(\gamma _3)$ spectra. Components $\mathrm {O(1)}$, $\mathrm {O(2)}$, $\mathrm {O(3)}$, and $\mathrm {O(4)}$ originate from O atoms in the lattice ($\mathrm {O_L}$), $\mathrm {O_LH}^-$ species, $\mathrm {CO_x}$ species, and $\textrm{OH}^-$ and/or $\mathrm {O_2}^-$ species, respectively. The ratio of component $\mathrm {O(2)}$ [$\mathrm {O(4)}$] over component $\mathrm {O(1)}$ as a function of exit angle range $\gamma$ is displayed in panel (**d**) [(**e**)] for the three samples under study.

O 1s PE spectra measured at different exit angle ranges $$\gamma$$ (Fig. 5a–c) provide a detailed picture of oxygen-related species at the BTO surface. Let us focus first on the most bulk sensitive $$\textrm{O}(\gamma _3)$$ spectra. Here, the most prominent peak $$\mathrm {O(1)}$$ at 529.8 eV refers to O atoms in BTO lattice ($$\mathrm {O_L}$$). The other O components result from $$\hbox {H}_2$$O dissociation ($$\mathrm {O(2)}$$ and $$\mathrm {O(4)}$$) and $$\mathrm {CO_x}$$ species ($$\mathrm {O(3)}$$). In general, adsorption of $$\mathrm {H_2O}$$ on BTO may lead to molecular physisorption with $$\mathrm {\Delta BE}\approx$$ 3.9 eV^64,65^. O 1s spectra in Fig. 5a–c show evidence of molecular physisorbed water only at the exit angle ranges $$\gamma _1$$ and $$\gamma _2$$ and with minor contributions (smaller than $$3\%$$) to the total spectral area. On the BaO termination, upon adsorption, water dissociates into $$\hbox {OH}^-$$ and $$\hbox {H}^+$$^12,13,67^, while on the $$\hbox {TiO}_2$$ termination both molecular and dissociated water may occur^12,64^. While $$\hbox {OH}^-$$ chemisorbs on top of cations (Ba or Ti) or at O vacancies^12–15^, $$\hbox {H}^+$$ binds to $$\mathrm {O_L}$$ at the BTO surface or diffuses inside the film to form $$\mathrm {O_LH}^-$$^14–16,63^, which is assigned to component $$\mathrm {O(2)}$$. The latter has a smaller binding energy shift $$\Delta \textrm{BE} =$$ 1 eV^68^, given the chemical environment closer to $$\mathrm {O_L}$$, as compared to chemisorbed $$\hbox {OH}^-$$. In contrast, $$\hbox {OH}^-$$ is assigned to component $$\mathrm {O(4)}$$ with a larger binding energy shift $$\Delta \textrm{BE} =$$ 2.7 eV^14,15^. In addition, other species, resulting from $$\mathrm {O_2}$$ adsorption such as peroxo complexes (e.g. $$\hbox {BaO}_2$$, Ti-O-O-Ti, Ti=$$\hbox {O}_{2}^{-}$$), can contribute to component $$\mathrm {O(4)}$$^64^. In this case, oxidation of BaO-terminated surfaces or Ti-OH leads to the presence of $$\hbox {O}_{2}^{-}$$ at the surface. Finally, component $$\mathrm {O(3)}$$ at $$\Delta \textrm{BE} = 1.8\hbox { eV}$$ is assigned to $$\hbox {CO}_\textrm{x}$$ species, such as carbonates $$\textrm{CO}_3^{2-}$$, $$\mathrm {C=O}$$ bonds, ester (C-(C=O)-OR) or carboxylic acid (C-(C=O)-OH) compounds^65,69^. In the BTO/SRO/DSO, BTO/SRO/GSO, and BTO/SRO/SSO samples, the contribution of component $$\mathrm {O(3)}$$ to the $$\gamma$$-integrated spectral area is $$\approx 0 \%$$, $$3 \%$$, and $$6 \%$$, respectively. Following the same substrate order, component $$\mathrm {O(2)}$$ [$$\mathrm {O(4)}$$] represents $$15 \%$$ [$$32 \%$$], $$19 \%$$ [$$30 \%$$], and $$13 \%$$ [$$53 \%$$] of the $$\gamma$$-integrated spectral area.

To gain information on the depth distribution of the different oxygen species, a quantitative analysis of $\mathrm {O(2)}$ and $\mathrm {O(4)}$ relative spectral area as a function of exit angle range $\gamma$ was performed. The ratio of spectral areas between components $\mathrm {O(2)}$ [$\mathrm {O(4)}$] and $\mathrm {O(1)}$, measured at exit angle range $\gamma _j$ ($j = 1, 2, 3$), is calculated as $R_2 = A_{\mathrm {O(2)}}^{\gamma _j} / A_{\mathrm {O(1)}}^{\gamma _j}$ [$R_4 = A_{\mathrm {O(4)}}^{\gamma _j} / A_{\mathrm {O(1)}}^{\gamma _j}$]. Figure 5d,e shows $R_2$ and $R_4$ at the different $\gamma _j$ together with the corresponding fit functions. The model employed to fit these data^70^ assumes that adsorbates form a patched overlayer of thickness $t_{\mathrm {O(k)}}$ ($k = 2, 4$) and coverage $\Gamma$ ($0<\Gamma <1$), which indicates the fraction of surface covered by the overlayer. $R_2$ and $R_4$ data in Fig. 5d,e are fitted with the fitting parameter $\Gamma$, while $t_{\mathrm {O(k)}}$ is varied in 1 Å steps to obtain the best fit with $R^2 \approx 1$. On all samples, the adsorbates represented by component $\mathrm {O(4)}$ form a superficial layer of thickness $\approx$ 4 Å, corresponding to 1 monolayer^64^. In contrast, in the BTO/SRO/DSO sample, component $\mathrm {O(2)}$ is distributed below the BTO surface with thickness $\approx$ 15 Å, while in the BTO/SRO/GSO and BTO/SRO/SSO samples, component $\mathrm {O(2)}$ is concentrated near the surface with thickness $\approx$ 6 Å. The larger thickness $t_{\mathrm {O(2)}}$ in the BTO/SRO/DSO sample cannot be explained by a thicker overlayer of $\mathrm {O_LH}^-$ species above the BTO surface because the molecules in the overlayer would be in a different chemical environment and thus appear at a different binding energy. Instead, our experimental data suggest a scenario where $\textrm{H}^+$ atoms are distributed below the BTO surface to form $\mathrm {O_LH}^-$ species (Fig. 7b), as already proposed in other works^15,16^.

### Ba and Ti XSW

**Figure 6:**
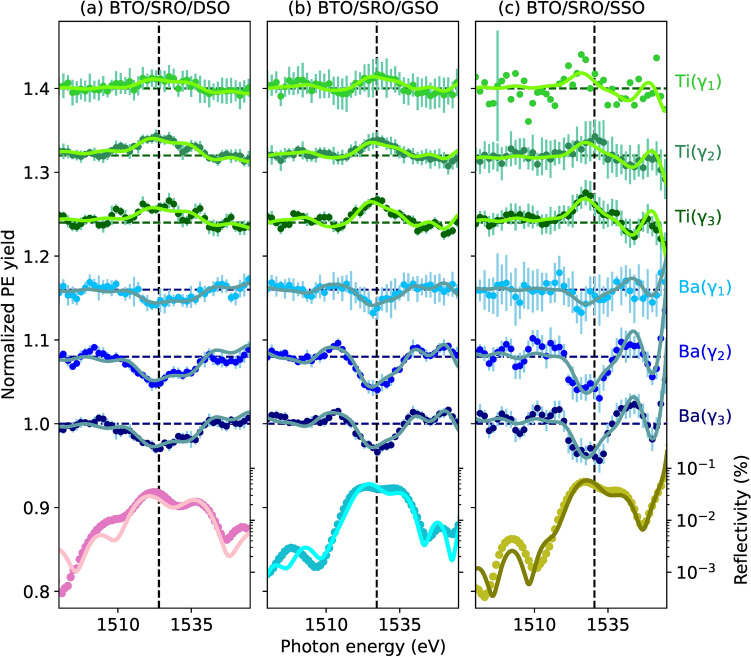
Ti and Ba PE yield data (green and blue points) measured at the exit angle ranges $\gamma _1$, $\gamma _2$, and $\gamma _3$ on BTO/SRO/DSO (**a**), BTO/SRO/GSO (**b**), and BTO/SRO/SSO (**c**), and corresponding fit curves (solid lines). Reflectivity $R_0(E\nu )$ data and fit curves around the (001) BTO Bragg energies $E_B =$ 1524 eV (**a**), 1527.2 eV (**b**), 1530.4 eV (**c**) (marked by vertical dashed lines). For clarity, $\kappa ^{\textrm{Ti}}_{\gamma _1}(E_\nu )$ of BTO/SRO/SSO is shown with only one error bar, which corresponds to the average error bar of all $\kappa ^{\textrm{Ti}}_{\gamma _1}(E_\nu )$ data points. All PE yield curves are normalized (see Supplementary Note 5) and, for clarity, the curves above $\textrm{Ba}(\gamma _3)$ are vertically shifted by 0.08 from the one below.

Figure 6 shows the normalized PE yields of Ti and Ba, $\kappa ^{\textrm{Ti}}_\gamma (E_\nu )$ and $\kappa ^{\textrm{Ba}}_\gamma (E_\nu )$, measured over the exit angle ranges $\gamma _1$, $\gamma _2$, and $\gamma _3$ around the BTO (001) Bragg peak of the three samples under study. Each $\kappa ^s_\gamma (E_\nu )$ and corresponding error bar $\sigma _\kappa$ result from the average and standard deviation of *N* photoelectron yield profiles ($5<N<10$) measured under the same conditions. Each PE yield curve shown in Fig. 6 is normalized by the intensity of the incoming X-ray beam and by the respective photoionization cross section over the measured photon energy range (see Supplementary Note 5). In correspondence to the BTO reflectivity maxima, $\kappa ^{\textrm{Ti}}_\gamma (E_\nu )$ curves show a peak-like shape, while $\kappa ^{\textrm{Ba}}_\gamma (E_\nu )$ profiles display a dip. This can be explained as follows. For the BTO (001) reflection, the Bragg diffraction planes are near the Ba atomic planes^42,71^ (solid lines in Fig. 1b). When the incoming X-ray photon energy reaches the Bragg condition ($E_\nu \approx E_B$) from the low-energy side, the XSW forms with a sinusoidal modulation of the X-ray intensity $I_{\textrm{XSW}}$ and period $d_{001}$ along $\varvec{H}$. At this point, the standing wave antinodes and nodes are between and at the diffraction planes, respectively (Fig. 1a,b). Therefore, Ti atoms, which are nearly half way between two adjacent diffraction planes and hence more aligned with the antinodes, show an increase in the PE yield, while Ba atoms (near the diffraction planes and aligned with the nodes) experience a decrease in $I_\textrm{XSW}$ and consequently have smaller $\kappa ^{\textrm{Ba}}_\gamma (E_\nu )$. As the photon energy is varied through the Bragg condition ($E_\nu >E_B$), the nodes and antinodes move by $d_{001}/2$ along $\varvec{H}$ and the XSW intensity modulation fades away. Because of the weak diffraction of the incoming X-ray wave from the thin film, the reflectivity maxima of our samples range from $0.03\%$ to $0.06\%$. Therefore, the interference between the incoming and Bragg-diffracted X-ray waves results in a weak XSW intensity modulation with an amplitude, which is proportional to $2\sqrt{R_0(E_\nu )}$, of less than $4\%$. Nevertheless, as shown below, this is sufficient to determine, from the information encoded in the PE yield profiles, the average atomic distribution within the unit cell with picometer spatial accuracy.

**Table 2:** Coherent position $P_{c, \gamma }^s$ and coherent fraction $F_{c, \gamma }^s$ of Ba and Ti PE yield fits (Fig. 6) at the exit angle ranges $\gamma _1$, $\gamma _2$, and $\gamma _3$, in the three samples under study. Coherent position offset of Ti atoms from the center of the unit cell defined by Ba atoms, $\Delta P^{\textrm{Ti}}_{c,\gamma } = P^{\textrm{Ti}}_{c,\gamma } - \left( P^{\textrm{Ba}}_{c,\gamma }-0.5\right)$, and absolute off-center displacement of Ti atoms, $\Delta z^{\textrm{Ti}}_\gamma = c_0 \Delta P^{\textrm{Ti}}_{c,\gamma }$, expressed in picometer.

Sample	Angle range	$P_{c, \gamma }^{\textrm{Ba}}$	$F_{c, \gamma }^{\textrm{Ba}}$	$P_{c, \gamma }^{\textrm{Ti}}$	$F_{c, \gamma }^{\textrm{Ti}}$	$\Delta P^{\textrm{Ti}}_{c,\gamma }$	$\Delta z^{\textrm{Ti}}_\gamma$ (pm)
BTO/SRO/DSO	$\gamma _1$	1.10(1)	0.50(7)	0.64(3)	0.35(9)	0.04(4)	16
BTO/SRO/DSO	$\gamma _2$	1.11(1)	0.88(6)	0.64(2)	0.56(7)	0.03(3)	12
BTO/SRO/DSO	$\gamma _3$	1.11(1)	0.87(8)	0.63(2)	0.48(8)	0.02(3)	8
BTO/SRO/GSO	$\gamma _1$	1.05(1)	0.47(5)	0.54(3)	0.29(6)	-0.01(4)	-4
BTO/SRO/GSO	$\gamma _2$	1.06(1)	0.88(3)	0.55(2)	0.32(5)	-0.01(3)	-4
BTO/SRO/GSO	$\gamma _3$	1.04(1)	0.78(3)	0.56(1)	0.54(5)	0.02(2)	8
BTO/SRO/SSO	$\gamma _1$	1.08(2)	0.42(6)	0.63(6)	0.42(18)	0.05(9)	20
BTO/SRO/SSO	$\gamma _2$	1.02(1)	1.00(11)	0.55(4)	0.39(12)	0.03(5)	12
BTO/SRO/SSO	$\gamma _3$	1.02(1)	0.99(7)	0.56(2)	0.58(9)	0.04(3)	16

**Figure 7:**
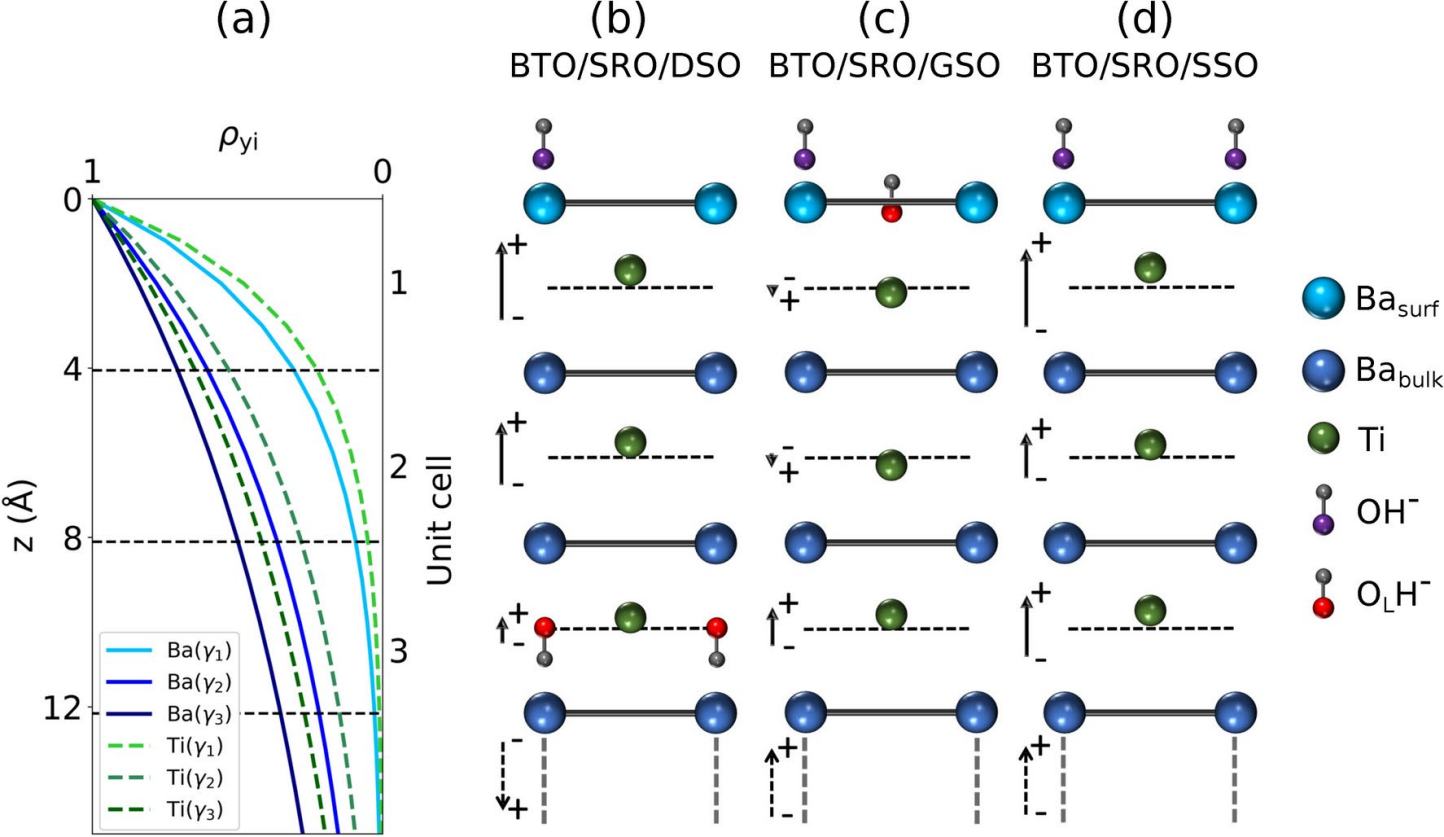
(**a**) Probability yield functions $\rho _\textrm{yi}(z)$ of Ba 4d and Ti 2p photoelectrons at $E_B =$ 1525 eV integrated over the three exit angle ranges $\gamma _1$, $\gamma _2$ and $\gamma _3$. Sketch of the top three unit cells of BTO/SRO/DSO (**b**), BTO/SRO/GSO (**c**), and BTO/SRO/SSO (**d**). Note that Ti atomic displacements $\Delta z^{\textrm{Ti}}_\gamma$ displayed in the sketch do not refer to the atomic positions at the corresponding unit cell, but rather to the $\rho _\textrm{yi}(z)$-weighted average of atomic positions in different unit cells with contributions calculated as detailed in “Methods”, “Depth-dependent photoelectron contribution”. For a better visualization, Ti atomic displacements $\Delta z^{\textrm{Ti}}_\gamma$ are twice larger than values in Table 2. The length of polarization vectors (solid arrows) is proportional to the corresponding $\Delta z^{\textrm{Ti}}_\gamma$. The direction of the average ferroelectric polarization in BTO films measured by PFM is marked by dashed arrows below the third unit cell. For clarity, only $\textrm{OH}^-$ and $\mathrm {O_LH}^-$ species are sketched, while $\mathrm {O_2}^-$ species and $\mathrm {O_L}$ atoms are omitted. In panel (**b**) the orientation of the $\mathrm {O_LH}^-$ group is one of the possible stable configurations^16^. The present sketch indicates, as an example, only BaO termination, however our samples have mixed (BaO and $\hbox {TiO}_2$) termination (see “Methods”, “Sample growth”), and a similar sketch can be drawn for $\hbox {TiO}_2$ termination.

The XSW analysis presented here is based on the calculation of the reflectivity $R_0(E_\nu )$ which assumes either upward or downward average polarization of the BTO film, as it results from PFM data (Fig. 2d–f and Table 1). The respective positions of Ba, Ti and O atoms in the unit cell for the reflectivity calculations come from known BTO bulk values^5^. The model employed is validated by the reasonably good fit of both reflectivity and yield data. In fact, the experimental PE yields shown in Fig. 6 are well fitted by Eq. (3) with the fitting results, $P^s_{c,\gamma }$ and $F^s_{c,\gamma }$, summarized in Table 2. As expected from the atomic coordinates used to construct the structural model in the XSW analysis, Ba atoms have $P^{\textrm{Ba}}_{c,\gamma }\approx 1$, while Ti atoms have $P^{\textrm{Ti}}_{c,\gamma }\approx 0.5$. Their exact atomic positions vary with sample and depth by up to few tens of picometer with an error bar (averaged over $\gamma$) of 4 pm for Ba and 12 pm for Ti (see “Methods”, “The uncertainty of Ba and Ti atomic positions”). In the context of a displacive ferroelectric like BTO^72^, the relevant physical quantity is the displacement of Ti atoms from the center of the unit cell (defined by $P^{\textrm{Ba}}_{c,\gamma }$), which directly relates to the ferroelectric polarization^73^. Therefore, we calculate $\Delta P^{\textrm{Ti}}_{c,\gamma } = P^{\textrm{Ti}}_{c,\gamma } - \left( P^{\textrm{Ba}}_{c,\gamma }-0.5\right)$ and the corresponding absolute off-center displacement (in picometer) of Ti atoms $\Delta z^{\textrm{Ti}}_\gamma = c_0 \Delta P^{\textrm{Ti}}_{c,\gamma }$. XSW data at different exit angle ranges $\gamma$ provide Ti atomic displacements at different depths *z* from the BTO surface. For Ba 4d [Ti 2p] photoelectrons at the (001) BTO Bragg energy $E_B$, the escape depths are $\lambda _{l,\gamma } =$ 3.4 Å, 8.0 Å, 11.6 Å [2.7 Å, 6.4 Å, 9.2 Å] for $\gamma _1$, $\gamma _2$ and $\gamma _3$, respectively (see Section X-ray standing waves generated in thin films and “Methods”, “Inelastic mean free path”). The corresponding probability yield functions $\rho _\textrm{yi}(z)$, integrated over the three exit angle ranges $\gamma _1$, $\gamma _2$ and $\gamma _3$ (Fig. 7a), indicate that $\Delta z^{\textrm{Ti}}_{\gamma _1}$ relates mostly ($\approx 70\%$) to atoms within the first unit cell, while $\Delta z^{\textrm{Ti}}_{\gamma _2}$ [$\Delta z^{\textrm{Ti}}_{\gamma _3}$] results primarily from a $\rho _\textrm{yi}(z)$-weighted average of atomic positions within the top 2 [3] unit cells. The remaining ($\approx 30\%$) contribution comes from the $\rho _\textrm{yi}(z)$-weighted average of atomic positions underneath (see “Methods”, “Depth-dependent photoelectron contribution”). Figure 7b–d shows a sketch of Ti atomic displacements $\Delta z^{\textrm{Ti}}_{\gamma }$ (Table 2) mapped for simplicity to the top three BTO unit cells to highlight their depth-dependence. In particular, the XSW fit results of the BTO/SRO/DSO sample reveal positive $\Delta z^{\textrm{Ti}}_\gamma$ values which decrease as $\gamma$ increases. This corresponds to an upward ferroelectric polarization $\textrm{P}^\uparrow$ with decreasing amplitude from the surface to the bulk. In contrast, the BTO/SRO/GSO sample shows a minor offset $\Delta z^{\textrm{Ti}}_\gamma < 0$ for $\gamma _1$ and $\gamma _2$, while $\Delta z^{\textrm{Ti}}_{\gamma _3} > 0$. This indicates an upward polarization $\textrm{P}^\uparrow$ at larger depths that nearly vanishes with a minor reversal just below the surface. Finally, in the BTO/SRO/SSO sample, $\Delta z^{\textrm{Ti}}_{\gamma } > 0$, i.e., an upward polarization ($\textrm{P}^\uparrow$), is observed for all $\gamma$.

It is important to highlight that the atomic positions provided by the XSW technique refer to the Bragg diffraction planes, which, in turn, depend on the positions of each atom in the unit cell. Specifically, it has been shown^42,71^ that the XSW phase is directly linked to the phase $\beta _h$ of the structure factor ${F_h = \left| F_h\right| \exp (\textrm{i}\beta _h) = \sum_j f_{h}^{j} \exp (-W_j^\textrm{T}) \exp (-\textrm{i} \boldsymbol{h} \boldsymbol{r}_j )}$. Here, $f_j$ and $\boldsymbol{r}_j$ are the atomic scattering factors and atomic positions of the *j*th atom, respectively, and $\exp (-W_j^\textrm{T})$ is the thermal Debye–Waller factor (“Methods”, “Reflection and transmission calculation”). In the presence of a deformation field, as in our case, also the deformation phase $\varphi _0$ should be considered. As a result, the absolute position of the Bragg diffraction planes with respect to the origin of the unit cell, here arbitrarily taken as the Ba atom, is given by $c(\beta _h + \varphi _0)/(2\pi )$. This implies that the position of a given atom, obtained from the XSW technique, depends on the position of the diffraction planes, and thus on the structure of the unit cell. For example, as it was already observed in $\textrm{PbTiO}_3$ for Pb atoms^42^, also in BTO, Ba atoms in $\textrm{P}^\uparrow$ and $\textrm{P}^\downarrow$ configurations have different positions with respect to the diffraction planes because of the different position of O and Ti atoms and thus of the diffraction planes with respect to the lattice (Fig. 1b). As a consequence, rather than focusing on the individual Ba and Ti positions, considering their relative position $\Delta z^{\textrm{Ti}}_{c,\gamma }$ (defined above) is more useful and relevant to discuss local polarization changes.

We move now to discussing the atomic coherent fractions. For Ba at the exit angle ranges $\gamma _2$ and $\gamma _3$ the coherent fraction is relatively high in all samples ($F^{\textrm{Ba}}_{c,\gamma } > 0.8$), indicating high structural order. In particular, in BTO/SRO/SSO, where no reversal of ferroelectric polarization with depth is observed, $F^{\textrm{Ba}}_{c,\gamma _2}$ and $F^{\textrm{Ba}}_{c,\gamma _3}$ are equal to 1. The latter values are an overestimation because at room temperature atomic vibrations lead to $F_{c} < 1$, even in a perfectly ordered atomic layer. The overestimation of $F_{c}$ is due to two possible reasons. First, our XSW analysis does not include non-dipolar parameters, which are currently not available for p, d, and f initial states^74^, and thus, higher $F_{c}$ are expected without correcting for non-dipole effects. Second, the nonlinear behaviour of the MCP may lead to an overestimation of the count rate, consequently of the XSW modulation amplitude, and thus of $F_{c}$. Conversely, in BTO/SRO/DSO and BTO/SRO/GSO, $F^{\textrm{Ba}}_{c,\gamma _2}$ and $F^{\textrm{Ba}}_{c,\gamma _3}$ values are $12\%$ to $22\%$ lower, depending on sample and $\gamma$. This is attributed to the averaging over atoms in unit cells with different polarizations, which also contribute to the generally lower coherent fraction of Ti atoms in the range of $0.3{-}0.6$. On the other hand, for the most surface sensitive measurements at $\gamma _1$, both Ba and Ti atoms have a lower coherent fraction of 0.3 to 0.5. The generally lower $F^{s}_{c,\gamma }$ at the surface can be attributed to the larger structural disorder induced by the interactions of the atoms at the topmost oxide plane with adsorbates. Furthermore, we note that the structural accuracy of the XSW technique results from the X-ray standing wave formed in the thin film, therefore it is not affected by the surface roughness of $S_\textrm{RMS} = 0.84(8)\hbox { nm}$. In fact, the latter accounts for only $2-4\%$ of the BTO thickness. In general, in comparison to an atomically flat surface, a larger surface roughness provides a larger surface area where various species may adsorb. In our samples, $S_\textrm{RMS}$ is approximately the same for all the samples, thus differences in atomic positions cannot be attributed to the surface roughness.

As presented in the previous section, the components of Ba 4d core level originating from surface and bulk species can be differentiated. The respective XSW yield profiles were fitted (Supplementary Fig. 8) and the resulting structural parameters ($P^{\textrm{Ba}}_{c,\gamma _1}$, $F^{\textrm{Ba}}_{c,\gamma _1}$) are reported in Table 3. The comparison is limited to the angle range $\gamma _1$ because the $\mathrm {Ba_{surf}}$ component refers to Ba atoms at the topmost layer that have the largest contribution to XPS spectra at the most grazing exit angle $\gamma _1$. The first observation, valid for all samples, is that the coherent fraction of the surface component is relatively low, below 0.3, and approximately $3-11$ times smaller compared to the one of the bulk component. This is due to the larger disorder at the surface induced by the presence of adsorbates. In fact, the lowest $F_c$ correlates well with the largest amount of adsorbates on the BTO/SRO/SSO sample. Because of the generally low $F_c$ of the surface component, the corresponding $P_c$ is less relevant since it refers to an average over atoms with a larger positional spread. Nevertheless, some trends are observed. Specifically, in the BTO/SRO/DSO sample $P_{c, \textrm{surf}} < P_{c, \textrm{bulk}}$, while in the other two samples $P_{c, \textrm{surf}} > P_{c, \textrm{bulk}}$, where a larger $P_c$ indicates a larger distance of Ba atoms from the Bragg diffraction planes. In addition, $P_{c, \textrm{bulk}}$ of sample BTO/SRO/DSO is larger than in the other two samples, while the opposite is true for $P_{c, \textrm{surf}}$. These trends can be understood by looking at the distribution of adsorbates at the surface (Section Ba, Ti and O XPS) and at available DFT calculations^11^. The latter predict that the hydroxyl group $\textrm{OH}^-$ and O adatoms tend to pull Ba atoms up, i.e., away from the substrate, while H adatoms tend to push Ba atoms down. In the BTO/SRO/DSO sample, the distribution of interstitial H atoms below the surface forms $\mathrm {O_L H}^-$ groups that lift Ba atoms in the bulk further away from the diffraction planes, as compared to those at the surface. This is confirmed by the comparison with the other samples, where, in the absence of H diffusion, Ba positions are lower (Table 3). In the BTO/SRO/GSO sample, the presence of both $\textrm{OH}^-$ and $\textrm{H}^+$ adsorbates with opposite tendencies leads to the smallest deviation between surface and bulk positions of Ba atoms. In the BTO/SRO/SSO sample, the largest concentration of $\textrm{OH}^-$ or $\mathrm {O_2}^-$ species leads to the largest average distance of Ba atoms at the surface from the diffraction planes, as compared to the bulk Ba atoms. To determine more accurately the position of Ba atoms near the surface, the Ba data at the smallest exit angle range $\gamma _1$ in Table 2 refer to the sum of surface and bulk components. On the other hand, Ba data at angle ranges $\gamma _2$ and $\gamma _3$ refer only to the bulk component, in order to exclude the contribution of Ba surface atoms and obtain atomic positions more representative of the unit cells below the surface.

**Table 3:** Coherent position $P_{c}$ and coherent fraction $F_{c}$ of surface and bulk components of Ba atoms measured at the angle range $\gamma _1$, in the three samples under study.

	BTO/SRO/DSO		BTO/SRO/GSO		BTO/SRO/SSO	
Component	$P_{c}$	$F_{c}$	$P_{c}$	$F_{c}$	$P_{c}$	$F_{c}$
surface	1.02(1)	0.17(61)	1.08(2)	0.28(5)	1.24(8)	0.09(8)
bulk	1.11(1)	0.64(11)	1.03(1)	0.71(6)	1.05(1)	0.99(9)

In addition, we attempted to apply the XSW analysis also to O 1s components, only on the BTO/SRO/DSO sample due to the limited time for performing the experiments. However, the low signal level, due to the lower photoionization cross section^75,76^, and the low statistics led to large error bars in the PE yield profiles, preventing a reliable determination of the corresponding structural parameters ($P^s_{c,\gamma }$, $F^s_{c,\gamma }$).

Finally, given the structural model resulting from the fit of the reflectivity data, the XSW yield fit curves can describe well the overall trend of the experimental yield profiles. A careful inspection of the yield curves reveals the presence of oscillations with a periodicity of $\approx 10\hbox { eV}$. These are relatively clear in the Ti($\gamma _3$) data of the BTO/SRO/DSO and BTO/SRO/GSO samples. We discuss next the possible origin of these features. First, oscillations of the incident X-ray intensity can be excluded, because each data point is normalized by the corresponding X-ray intensity $\hbox {I}_0$, as detailed in Supplementary Note 5. In addition, Supplementary Fig. 7b displays few typical $\hbox {I}_0$ profiles and there are no oscillations visible. Second, in general, photoelectron diffraction and extended X-ray absorption fine structure (EXAFS) show intensity oscillations as a function of photon energy. However, these effects can be safely excluded here, because the oscillation period of $\approx 10\hbox { eV}$ would result in unphysically large bond lengths. In fact, typical oscillation periods are of the order of $50{-} 100\hbox { eV}$^77^. Third, oscillations resulting from beam interferences due to a small sample thickness have the same periodicity both in the reflectivity and the yield curves. Hence, this cannot explain the additional smaller periodicity visible in some of our data. Fourth, it is well known that the deformation field in a crystal can lead to the occurrence of oscillations in both the reflectivity and the yield curves^46^. This is the case when the deformation extends throughout the sample, e.g., as in the case of sample bending^78^. Conversely, if the deformation field affects only a smaller portion of the sample, then the reflectivity will not be sensitive to this. On the other hand, if the near-surface region is affected, the photoelectron yield will be able to probe it and oscillations with frequency higher than the ones of the reflectivity may appear. Therefore, we assign the visible oscillations in the yield curves to the effect of a deformation field near the surface. The origin of this is likely a more complex strain distribution, which is beyond our simple structural models that capture the main features of the diffraction data, but not every detail. Furthermore, it is not surprising that such fine oscillations in the yield curves are not reproduced by the present models, because the latter are based on the reflectivity data, where the higher frequency oscillations are not visible.

## Discussion

XSW data reveal that the absolute displacements of Ti atoms from the center of the unit cell ($\Delta z^{\textrm{Ti}}_{\gamma _i}$) decrease from BTO/SRO/SSO, through BTO/SRO/DSO, to BTO/SRO/GSO. This trend is not correlated with the in-plane compressive strain $\varepsilon ^{a}_\textrm{BTO}$, as it could have been expected. Instead, we explain the measured off-center displacements of Ti atoms in light of oxygen-containing adsorbates at the surface, as detailed below. Upon exposure to ambient conditions water adsorbs on the BTO surface and dissociates into $\hbox {OH}^-$ and $\hbox {H}^+$. While $\hbox {OH}^-$ chemisorbs on top of cations (Ba or Ti) or at O vacancies^12–15^, $\hbox {H}^+$ binds to a lattice oxygen atom $\left(\mathrm {O_L}\right)$ at or below the surface to form $\mathrm {O_LH}^-$^14–16,63^. In summary, our depth-dependent O 1s XPS spectra reveal the presence of: (i) negatively charged chemisorbed O species, i.e., $\textrm{OH}^-$ (hydroxyl groups) or $\mathrm {O_2}^-$ (peroxo groups), modeled by the component $\mathrm {O(4)}$, and (ii) $\mathrm {O_LH}^-$ species, resulting from a $\textrm{H}^+$ ion bound to an $\mathrm {O_L}$, or a hydroxyl group adsorbed at a oxygen-vacancy site, modeled by the component $\mathrm {O(2)}$. DFT calculations on the similar ferroelectric $\hbox {PbTiO}_3$^11^, predict that, regardless of the surface termination, negatively charged $\textrm{OH}^-$ or $\mathrm {O_2}^-$ molecules favor the upward polarization $\textrm{P}^\uparrow$, while positively charged $\textrm{H}^+$ atoms of $\mathrm {O_LH}^-$ species favor the downward polarization $\textrm{P}^\downarrow$.

In the BTO/SRO/DSO sample, the BTO film has downward average polarization $\textrm{P}^\downarrow$ (Table 1) with upward polarization $\textrm{P}^\uparrow$ in the topmost unit cells (Fig. 7b). The latter is favored by negatively charged $\textrm{OH}^-$ or $\mathrm {O_2}^-$ molecules adsorbed on the surface, represented by component $\mathrm {O(4)}$ in Fig. 5a. In particular, the off-center displacement of Ti atoms decreases from the surface towards the bulk. This trend is consistent with a reversal of the ferroelectric polarization below the top three unit cells, which however is beyond our XSW depth sensitivity. For this configuration to be stable, a concentration of positive charges at the polarization flip interface is required. Importantly, the depth dependence of component $\mathrm {O(2)}$ in this sample is consistent with a distribution of $\mathrm {O_LH}^-$ species over about 4 unit cells ($\approx$ 15 Å) below the surface (Fig. 5d), and thus indicates the accumulation of $\textrm{H}^+$ atoms as a possible charge compensation mechanism for the polarization reversal below the surface (Fig. 7b). A similar scenario has been suggested by Lee and coworkers^15^. In their study, atomic positions across the BTO thin film were derived from CTR scattering experiments, while the increase in component $\mathrm {O(2)}$ upon water adsorption was assigned to the presence of $\textrm{H}^+$ or defects. In our study, the combination of depth-dependent XSW and XPS, and PFM provides further experimental evidence that suggests the presence of a $\textrm{H}^+$-mediated polarization reversal below the BTO surface.

In the BTO/SRO/GSO sample, the BTO film has an upward average polarization $\textrm{P}^\uparrow$ (Table 1) in agreement with a positive displacement of Ti atoms in the most bulk-sensitive data ($\Delta z^{\textrm{Ti}}_{\gamma _3} > 0$). In contrast, the more surface-sensitive data show a small negative displacement of Ti atomic positions (Fig. 7c), which indicates a minor polarization reversal at the top unit cells. In fact, in this sample, contrary to the one above, O 1s XPS data show that $\mathrm {O_LH}^-$ species are confined at the surface with concentration similar to $\textrm{OH}^-$ or $\mathrm {O_2}^-$ species (Fig. 5b,d). The minor displacement of Ti atoms from the center of the unit cells near the surface, resulting in a vanishing net polarization, is attributed to the competition between positively charged $\textrm{H}^+$ atoms of $\mathrm {O_LH}^-$ species that favor downward polarization $\textrm{P}^\downarrow$ and negatively charged $\textrm{OH}^-$ or $\mathrm {O_2}^-$ molecules that favor upward polarization $\textrm{P}^\uparrow$^11^.

In the BTO/SRO/SSO sample, the BTO film has an upward average polarization $\textrm{P}^\uparrow$ (Table 1), and a positive off-center displacement of Ti atoms throughout the top BTO unit cells is observed (Fig. 7d). This scenario implies upward polarization $\textrm{P}^{\uparrow }$ with accumulation of positive bound charge at the surface. To stabilize this configuration, a compensating negative screening charge at the surface is required. O 1s XPS spectra (Fig. 5c) show a large concentration of negatively charged O species ($\hbox {OH}^-$ and/or $\mathrm {O_2}^-$). DFT calculations^11^ predict that $\hbox {OH}^-$ adsorbates and $\mathrm {O_2}^-$ adatoms favor an upward polarization $\textrm{P}^\uparrow$, thereby inducing a larger offset of Ti atoms in the topmost atomic plane. Our XSW data show a larger displacement of Ti atoms at the surface (20 pm), as compared to deeper unit cells, and thus provide direct experimental evidence for this predicted behavior.

In single-domain BTO bulk crystals at room temperature with upward $\textrm{P}^{\uparrow }$ [downward $\textrm{P}^{\downarrow }$] polarization, neutron diffraction analysis revealed that the displacement of Ti atoms is 5 pm above [below] the center of the unit cell^5^. In comparison, the most bulk-sensitive XSW data $\Delta z^{\textrm{Ti}}_{\gamma _3}$ show larger Ti atomic displacements. This can be explained by the residual strain at the top sublayer, leading to out-of-plane lattice parameters $c_0$ larger than the bulk value $c_{b,\textrm{BTO}}$, and consequently larger atomic displacements. Moreover, as shown above, atomic positions near the surface are influenced by adsorbates, which may lead to smaller (BTO/SRO/GSO) or larger (BTO/SRO/DSO and BTO/SRO/SSO) atomic displacements depending on their type and content.

In summary, the three samples under study have different in-plane compressive strain and thickness (Table 1), however the corresponding strain gradients lead to a similar average out-of-plane lattice parameter $c_0$ in the top sublayer of the BTO/SRO/GSO and BTO/SRO/SSO samples and a slightly smaller one in the BTO/SRO/DSO sample. Upon exposure to ambient conditions each sample displays a different distribution of the ferroelectric polarization near the surface with the common result of screening the bulk polarization and stabilizing the ferroelectric thin film surface. The available data show that there is a correlation between the polarization profile at the top unit cells and the type and content of adsorbates on the surface. The interplay of available adsorbates and bulk ferroelectric polarization leads to the resulting distribution of local polarization near the surface. Further studies are required to elucidate to which extent the adsorption of external species influences or is influenced by the polarization below the surface.

## Conclusions

In this work, the XSW technique is successfully applied to BTO thin films to determine the near-surface displacement profile of Ti atoms from the center of the unit cell, defined by Ba atomic positions. In previous studies, the XSW technique in combination with XFS has been employed to determine the polarization orientation of the entire film ($\textrm{P}^\uparrow$ or $\textrm{P}^\downarrow$)^40–43,79–82^. Here, we have measured the photoelectron yield to determine the near-surface displacement of Ti atoms independently from the polarization in the bulk of the thin film. First, modelling X-ray diffraction data has provided the distribution of out-of-plane lattice parameters resulting from the epitaxial strain in our thin films. Second, the structural sensitivity of the X-ray standing wave combined with the chemical specificity, surface sensitivity and depth selectivity of X-ray photoelectron spectroscopy has provided Ti and Ba atomic positions at different depths with picometer spatial resolution. Since the Ba position defines the center of the unit cell, the measure of the Ti position gives direct access to the local ferroelectric polarization near the surface. The ferroelectric polarization in the top unit cells of the BTO samples under study has been interpreted with the help of depth-dependent O 1s XPS spectra. A detailed analysis of oxygen species adsorbed on the surface has suggested the possible charge compensation mechanisms that are consistent with the distribution of ferroelectric polarizations derived from XSW data. In particular, we have identified three different scenarios: (i) a polarization reversal from downward $\textrm{P}^\downarrow$ to upward $\textrm{P}^\uparrow$ polarization leading to a tail-to-tail polarization configuration near the third unit cell that could be stabilized by $\textrm{H}^+$ atoms diffused below the surface (Fig. 7b); (ii) a minor polarization reversal from upward $\textrm{P}^\uparrow$ to downward $\textrm{P}^\downarrow$ polarization above the third unit cell leading to a vanishing polarization, as a result of the competing presence of $\mathrm {O_LH}^-$ which favors downward polarization $\textrm{P}^\downarrow$ and $\textrm{OH}^-$ or $\textrm{O}^{2-}$ which favor upward polarization $\textrm{P}^\uparrow$ (Fig. 7c); (iii) a uniform upward polarization $\textrm{P}^\uparrow$ in the BTO film up to the surface accompanied by a large concentration of compensating negatively charged chemisorbed oxygen species (Fig. 7d).

The variety of the observed ferroelectric configurations demonstrates the complexity of these systems and underscores the importance of investigating them from different perspectives to gain a better understanding of the mechanisms that determine the ferroelectric polarization profile at the surface. The novelty of this work lies in the combination of structural and spectroscopic information, offered by the XSW technique, to provide at once a comprehensive picture of the surface ferroelectric polarization profile. This type of study can be applied to other interesting oxides and can be extended to a broader class of other technologically relevant materials, such as multiferroics^83^. Moreover, in the context of catalytic reactions at ferroelectric surfaces the determination and control of surface polarization and the interplay with adsorbates is crucial. To this end, *in operando* XSW investigations can guide material engineering towards more efficient catalysts^23,24^. Importantly, the few-picometer structural accuracy of XSW provides a rigorous test for benchmarking different theoretical models and thereby improving their predictive power^11,12^. Finally, we anticipate that the XSW technique can be employed to investigate the dynamics of ferroelectric polarization switching, specifically to simultaneously track structural and electronic changes of atoms in real time and corresponding measurements at X-ray free-electron laser facilities are in preparation.

## Methods

### Sample growth

Epitaxial bilayers BTO/SRO are grown on DSO, GSO, SSO substrates using pulsed laser deposition. The ceramic targets of SRO and BTO were 8 cm away from the substrates and ablated using a KrF excimer laser ($\lambda = 248\hbox { nm},$ fluence $5.4 \hbox {Jcm}^{-2}$, 2 Hz repetition rate). The deposition of SRO and BTO layer is conducted in $\hbox {O}_2$ atmosphere with oxygen partial pressure $\hbox {pO}_2 = 100\hbox { mTorr}$ and deposition temperature of 908 K and 973 K, respectively. Sample cooling with the rate of $3\hbox { K min}^{-1}$ is conducted in the environment of saturated $\hbox {O}_2$ ($\hbox {pO}_2$ = $10^4$ mTorr) to prevent the formation of oxygen vacancies.

Our rare earth (RE) scandate substrates $\hbox {REScO}_3$ did not undergo any etching process, thus a mixed REO and $\hbox {ScO}_2$ termination is expected^84,85^. Regardless of the substrate termination, because $\hbox {RuO}_2$ is extremely volatile, the expected termination of SRO layer is SrO^85,86^. Finally, the termination of the top BTO layer depends on the oxygen partial pressure $\hbox {pO}_2$ during deposition^87,88^. In our samples, at the deposition temperature of 973 K and $\hbox {pO}_2$ = 100 mTorr, BTO thin films have mixed termination BaO and $\hbox {TiO}_2$^87^.

### Grazing X-ray reflectivity

Grazing X-ray reflectivity data of as-grown samples, measured by a PANalytical X’Pert Pro diffractometer, are shown in Supplementary Fig. 1. The measured reflectivity $R_g(q)$ can be expressed as

5 $$\begin{aligned} R_g(q) = R_F(q)\Big |\frac{1}{\rho _s}\int _{-\infty }^{\infty }\frac{d\rho _e(z)}{dz}\exp {(-\textrm{i}qz)dz}\Big |^2, \end{aligned}$$

where $R_F$ and $\rho _s$ are the Fresnel reflectivity and the electron density of the substrate, respectively^89^. In Eq. (5) $R_g$ is expressed as a function of the wavevector transfer $q = (4\pi \sin {\theta })/\lambda$ , where $\theta$ is the incident angle of X-ray and $\lambda =$ 1.54 Å is the wavelength of Cu K$\alpha$ incident radiation. In practice, thin film thicknesses are determined as follows. First, $R_F$ is calculated using the Parratt formalism^90^. Second, the Fourier inversion of $R_g/R_F$ provides the autocorrelation of the derivative of the electron density as a function of *z*, i.e., $\rho '_e = d\rho _e(z)/dz$. This function displays peaks in correspondence of the interfaces, where $\rho '_e$ is largest, thereby providing the thicknesses of layers above the substrate.

### X-ray reciprocal lattice map

Figure 2a–c shows X-ray reciprocal lattice maps of the three samples around (-103) substrate Bragg peak, measured by a PANalytical X’Pert Pro diffractometer. The diffraction peaks of the substrates have very narrow intensity distribution, while the intensity distribution of BTO and SRO layers are weaker and broader. Reciprocal lattice parameters $Q_\textrm{x}$ and $Q_\textrm{z}$ of intensity peaks are related to the real space in-plane and out-of-plane lattice parameters, *a* and *c*, by the following relations: $a = -\lambda /(2Q_\textrm{x})$ and $c = (3\lambda )/(2Q_\textrm{z})$^91^. The values $Q_\textrm{x}$ and $Q_\textrm{z}$ of each diffraction peak are obtained by fitting the intensity distribution with a pseudo-Voigt function^92^.

### Piezoresponse force microscopy

Piezoresponse force microscopy in Dual AC Resonance Tracking (DART) mode^93^ was used to probe the polarization of as-grown samples and to prove that ferroelectric polarization can be switched by the application of positive or negative voltage between the PFM tip and the SRO electrode. Figure 2d-f show the PFM phase image of each sample after the application of a voltage to switch the polarization inside the marked gray boxes. A positive [negative] voltage of sufficient amplitude forces the polarization to be $\textrm{P}^\downarrow$ [$\textrm{P}^\uparrow$], with the corresponding PFM phase $0^{\circ }$ [$180^{\circ }$]. The PFM phase outside the gray boxes indicates the polarization of the as-grown sample. Therefore, Fig. 2d–f shows an average downward polarization $\textrm{P}^\downarrow$ in the BTO/SRO/DSO sample, while an average upward polarization $\textrm{P}^\uparrow$ in the BTO/SRO/GSO and BTO/SRO/SSO samples. Note also that PFM phase images show no indication of multiple domains in any of the samples. Furthermore, switching spectroscopy PFM (SS-PFM) was employed to measure hysteresis loops on each sample (see Supplementary Fig. 2).

### Reflection and transmission calculation

The X-ray diffracted intensity from the sublayer $L_i$ with thickness $t_i$ at photon energy $E_\nu$ and $z_i< z < z_i + t_i$ is calculated as^47^:

6 $$\begin{aligned} R(E_\nu , z) = \beta |Y|^2|r(E_\nu , z)|^2= \beta |Y|^2\Bigg |\frac{x_1-x_2 x_3 \exp (-\sigma \Delta z_i)}{1-x_3\exp (-\sigma \Delta z_i)}\Bigg |^2, \end{aligned}$$

where $$x_1 = -\Big (b+\sqrt{b^2-C_1^2}\Big )/C_1$$, $$x_2 = -\Big (b-\sqrt{b^2-C_1^2}\Big )/C_1$$, and $$x_3 = (x_1-r_{t_i})\exp (\sigma t_i)/(x_2-r_{t_i}),$$$$\sigma = 2\textrm{i}\sqrt{b^2-C_1^2}/L_{ex}$$, and $$\Delta z_i = z - z_i$$. The reflection amplitude at the bottom [top] of $$L_i$$ is defined as $$r_{t_i} = r(E_\nu , \Delta z_i = t_i)$$ [$$r(E_\nu , \Delta z_i = 0)$$], and *r* at the top of $$L_i$$ is then treated as the reflection at the bottom of layer $$L_{i-1}$$. Starting from the boundary condition of $$r(E_\nu , z)=0$$ at the bottom of the substrate, Eq. (6) is employed recursively to calculate the diffracted intensity at the top of $$L_0$$, i.e. $$R(E_\nu , 0)$$ (see Section X-ray standing waves generated in thin films).

Parameters appearing in Eq. (6) are summarized in the following. In particular, *b* is defined as $b = -y(E_\nu ) - \textrm{i}y_0 + y_\varphi (z)$, where $y(E_\nu ) = 2\sqrt{\beta }(\sin ^2{\theta _B})(E_\nu - E_B)/(E_B X_\textrm{r}) + \chi _{0\textrm{r}}(1+\beta )/(2\sqrt{\beta }X_\textrm{r})$, and $y_0 = (\chi _{0\textrm{i}}(1+\beta ))/(2\sqrt{\beta }X_\textrm{r})$, $y_\varphi (z) = (L_{ex}/2) d\varphi (z)/dz$. Here, $y(E_\nu )$ is a dimensionless parameter that indicates the energy deviation from the exact Bragg energy $E_B$ during an incident photon energy scan, $y_0$ represents the attenuation of X-ray intensity due to photoelectric absorption and $y_\varphi (z)$ indicates the shift of diffraction planes due to lattice deformation. In the latter equations, the geometry factor $\beta$ is defined as $\beta = \Gamma _{\varvec{0}} / |\Gamma _{\varvec{h}}|$, where $\Gamma _{\varvec{0}} = k_{\varvec{0}z}/K$ and $\Gamma _{\varvec{h}}=k_{\varvec{h}z}/K$ are the direction parameters with $K=2\pi /\lambda _B$ and $\lambda _B$ is the Bragg wavelength. The extinction length represents the penetration depth of the XSW field and is defined as $L_{ex} = (\lambda _B \Gamma _{\varvec{0}})/(\pi \sqrt{\beta }X_\textrm{r})$.

The parameter $C_1$ has the form $C_1=C(1-\textrm{i}p)\exp (-W(z))$, where $p=-X_\textrm{i}/X_\textrm{r}$ and *C* is the polarization factor, which is equal to 1 for $\sigma$ polarization and $\cos {2\theta _B}$ for $\pi$ polarization, with $\theta _B$ being the Bragg angle. The parameters $Y = \sqrt{\chi _{\varvec{h}}/\chi _{\bar{\varvec{h}}}} = |Y|\exp (\textrm{i}\Phi _Y)$, $X_{\textrm{r}}=Re\Big [\sqrt{\chi _{\varvec{h}} \chi _{\bar{\varvec{h}}}}\Big ]$ and $X_\textrm{i}=Im\Big [\sqrt{\chi _{\varvec{h}} \chi _{\bar{\varvec{h}}}}\Big ]$ are derived from crystal susceptibilities $\chi _{0}$, $\chi _{\varvec{h}}$, $\chi _{\bar{\varvec{h}}}$ corresponding to vectors $\varvec{0}*\varvec{h}$, $\varvec{h}$, and $-\varvec{h}$, with $\varvec{h} = 2\pi \varvec{H}$. The crystal susceptibility $\chi _{\varvec{h}}$ is generally a complex number $\chi _{\varvec{h}} = \chi _{\varvec{h}\textrm{r}} + \textrm{i}\chi _{\varvec{h}\textrm{i}}$, where $\chi _{\varvec{h}\textrm{r}} = -\Big (\textrm{e}^2\lambda _B^2/mc^2\pi \Omega \Big )F_{\varvec{h}\textrm{r}}$ represents X-ray elastic scattering, while $\chi _{\varvec{h}\textrm{i}} = \Big (\textrm{e}^2\lambda _B^2/mc^2\pi \Omega \Big )F_{\varvec{h}\textrm{i}}$ stands for X-ray absorption. The structure factor $F_h = \sum _j f_j \exp (-W_j^\textrm{T}) \exp (-\textrm{i}\varvec{h} \varvec{r}_j)$ is computed from the atomic scattering factor $f_j$ of *j*th atom at position vector $\varvec{r}_j$ in the unit cell, and the thermal Debye–Waller factor $\exp (-W_j^\textrm{T})$. The atomic scattering factor $f_j = f_0(\theta _B, \lambda _B, Z) - Z + f_1(0, \lambda _B, Z) + \textrm{i}f_2(0, \lambda _B, Z)$ describes the interaction between X-rays and atoms, where *Z* is the atomic number, $f_0$, $f_1$, and $f_2$ are tabulated in Refs.^94,95^.

Finally, besides the reflection amplitude, solving the Takagi–Taupin equations also provides the transmission amplitude through the sublayer $L_i$^47^:

7 $$\begin{aligned} T(E_\nu , z) = \exp [\textrm{i}\Phi (E_\nu )\Delta z_i/2]\Bigg (\frac{1-x_3\exp (-\sigma \Delta z_i)}{1-x_3} \Bigg ), \end{aligned}$$

where $$\Phi (E_\nu )=(2\pi \chi _{\varvec{0}})/(\lambda _B \Gamma _{\varvec{0}})- 2C_1 x_1/L_{ex}$$.

### Deformation phase calculation

The deformation phase $\varphi _0$ in Eq. (3), is derived from the bicrystal model^47^ and is defined as $\varphi _0 = 2 \pi ( c_0 - \overline{c})t_0/\overline{c}^2.$ The bicrystal model assumes: a crystal, in which the XSW is formed, and a deformed overlayer of thickness $t_l$ that induces a shift of the diffraction planes $\varphi _0 = 2 \pi ( c_l - c_c)t_l/c_c^2$, where $c_l$ and $c_c$ are the out-of-plane lattice parameters of the deformed layer and the crystal, respectively. In our samples, the XSW forms in the BTO film itself with a deformation given by an inhomogeneous strain. Therefore the deformation phase cannot refer to the *c* parameter of a single crystal underneath. Instead, $\varphi _0$ refers to the average out-of-plane lattice parameter $\overline{c}$, calculated from the experimental diffraction curves (see “Methods”, “[Sec Sec18]”). This is equivalent to modelling the thin film as a crystal with an average out-of-plane parameter $\overline{c}$ and an increasing [decreasing] deformation phase towards the interface with SRO [towards the surface]. The validity of this choice is confirmed by the coherent position of the Ba atoms (Table 2) being close to 1, i.e., near the diffraction planes. Conversely, referring, e.g., the deformation phase to the bottom sublayer $L_{n-1}$ would lead at the top unit cells to unphysical positions of the Ba atoms 1 Å away from the diffraction planes. The deformation phases $\varphi _0 / (2\pi )$ for samples BTO/SRO/DSO, BTO/SRO/GSO, and BTO/SRO/SSO, resulting from the fit of experimental (001) Bragg reflections, are 0.11, 0.08, and 0.04, respectively.

### Average out-of-plane lattice parameter $\overline{c}$

BTO and SRO average out-of-plane parameters $\overline{c}$ are calculated from the corresponding (001) Bragg peaks using the Bragg condition $\overline{c} = (12400\hbox { eV}{\text{\AA }})/(2\overline{E}_\nu \sin \theta _B)$. $\overline{E}_\nu$ is the average of energy values $E_\nu$ around the Bragg peaks, weighted with reflectivity $R_0(E_\nu )$. The energy ranges for calculating $\overline{E}_\nu$ of BTO and SRO are respectively 1510 eV–1547.5 eV and 1587 eV–1624 eV, where the $R_0(E_\nu )$ has finite values.

### Inelastic mean free path

The inelastic mean free path $\lambda _l(E_\nu )$ is defined, according to Ref.^96^, as:

8 $$\begin{aligned} \lambda _l(E_\nu ) = \frac{\mathscr {A}(E)(E_\nu - \textrm{BE})}{E_p^2\bigl \{\mathscr {B}\left[ ln(\mathscr {Y}\mathscr {A}(E_\nu )(E_\nu -\textrm{BE})) \right] -\mathscr {C}/(E_\nu -\textrm{BE})+\mathscr {D}/(E_\nu -\textrm{BE})^2 \bigr \}}. \end{aligned}$$

In Eq. (8): $\mathscr {A}(E_\nu ) = \left[ 1+(E_\nu -\textrm{BE})/(2m_ec^2)\right] /\left[ 1+(E_\nu -\textrm{BE})/(m_ec^2) \right] ^2$, $\mathscr {B} = -1.0 + 9.44/(E_p^2+E_g^2)^{0.5}+0.69\rho ^{0.1}$, $\mathscr {C} = 19.7-9.1\mathscr {U}$, $\mathscr {D} = 534-208\mathscr {U}$, $\mathscr {Y} = 0.191\rho ^{-0.5}$, and $\mathscr {U} = N_v\rho /M$. In these equations: $\textrm{BE}$ is the binding energy of photoelectrons from core level *l*, $m_e$ is the rest mass of electron, *c* is the speed of light, $N_v$ is the total number of valence electrons per molecule, $\rho$ is bulk density, *M* is the molecular weight, $E_p= 28.816( N_v.\rho /M )^{0.5}$ is the free-electron plasmon energy and $E_g$ is the band gap energy in eV. In the case of BTO: $N_v=24$, $M =233.19\hbox { g mol}^{-1}$^97^, $\rho = 6.02\hbox { gcm}^{-3}$^97^ and $E_g =$ 3.76 eV^98^. For Ba and Ti we consider $\textrm{BE}$ near the center of Ba 4d and Ti 2p PE spectra (Fig. 4), equal to 90 eV and 461 eV, respectively. As incident photon energy, we consider $E_\nu =$ 1527 eV, near the Bragg energy $E_B$ of the three samples. As a result, Eq. (8) provides $\lambda _\textrm{Ba 4d}(1527\hbox { eV}) =$ 25.3 Å and $\lambda _\textrm{Ti 2p}(1527\hbox { eV}) =$ 20.1 Å.

### Depth-dependent photoelectron contribution

The relative contribution of photoelectrons of atoms *s* emitted from a region between the surface $z_0 = 0$ and the depth $z_i = i \overline{c}_\textrm{BTO}$, and within the exit angle range $\gamma _j$ (with $j = 1, 2, 3$), is reported in Table 4 and calculated as follows:

9 $$\begin{aligned} I_{(z_0,z_i), \gamma _j}^{s} = (I_{\infty , \gamma _j}^{s})^{-1} \int _{z_0}^{z_i} \int _{\gamma _{j,l}}^{\gamma _{j,h}} \rho _\textrm{yi}\left( E_\nu ,z,\gamma \right) d\gamma dz, \end{aligned}$$

with $I_{\infty , \gamma _j}^{s}$ calculated as:

10 $$\begin{aligned} I_{\infty , \gamma _j}^{s} = \int _{z_0}^{\infty } \int _{\gamma _{j,l}}^{\gamma _{j,h}} \rho _\textrm{yi}\left( E_\nu ,z,\gamma \right) d\gamma dz, \end{aligned}$$

and $\rho _\textrm{yi}\left( E_\nu ,z,\gamma \right) = \exp \left( -z/\lambda _{l,\gamma }\right)$ (see Section X-ray standing waves generated in thin films). In the equations above, $\gamma _{i,l}$ and $\gamma _{i,h}$ are respectively the lower and higher limit of the exit angle range $\gamma _j$, reported in Table 4.

**Table 4:** Exit angle range $\gamma _j$, depth *z* range and corresponding relative contribution to the total Ba and Ti PE intensity.

*j*	($$\gamma _{i,l}$$, $$\gamma _{i,h}$$) ($$^\circ$$)	$$(z_0, z_i)$$$$\left[ (z_i, \infty )\right]$$	$$I_{(z_0,z_i), \gamma _j}^{\textrm{Ba}}$$$$\left[ I_{(z_i, \infty ), \gamma _j}^{\textrm{Ba}}\right]$$ ($$\%$$)	$$I_{(z_0,z_i), \gamma _j}^{\textrm{Ti}}$$$$\left[ I_{(z_i, \infty ), \gamma _j}^{\textrm{Ti}}\right]$$ ($$\%$$)
1	(2.4, 13.1)	($z_0$, $z_1$) [($z_1$, $\infty$)]	66 [34]	73 [27]
2	(13.1, 23.8)	($z_0$, $z_2$) [($z_2$, $\infty$)]	64 [36]	72 [28]
3	(23.8, 31)	($z_0$, $z_3$) [($z_3$, $\infty$)]	65 [35]	73 [27]

### The uncertainty of Ba and Ti atomic positions

The attainable structural accuracy in the determination of the average atomic positions depends ultimately on the error bar of the PE yield profiles according to Poisson statistics. We observe that the error bars of data of the BTO/SRO/SSO sample $\left(1\%< \sigma _\kappa < 7\%\right)$ are generally larger than those of the other two samples $\left(\sigma _\kappa < 2\%\right).$ This is due to the greater amount of adsorbates on the BTO/SRO/SSO surface, which attenuated the measured PE intensity (see Supplementary Note 2). Besides, lower PE intensity is also expected with a decreasing $\gamma$ or smaller $\lambda _{l,\gamma }$. This explains the trend of increasing $\sigma _\kappa$ with decreasing $\gamma$ and the larger uncertainty in Ti positions compared to Ba (Table 2). The latter observation is also due to the smaller photoionization cross section of Ti 2p as compared to Ba 4d core levels^75,76^.

## Electronic supplementary material

Below is the link to the electronic supplementary material.

Supplementary Information.

## Data Availability

The datasets used and/or analyzed during the current study are available from the corresponding author on reasonable request.
