# Supplementary material for: Surface polarization profile of ferroelectric thin films probed by X-ray standing waves and photoelectron spectroscopy
Source: Sci Rep. 2024 Oct 16;14:24250. doi: 10.1038/s41598-024-72805-1 (PMC11484970; doi:10.1038/s41598-024-72805-1)
Supplement: Supplementary file 1 — Supplementary Information. [file 41598_2024_72805_MOESM1_ESM.pdf]

Supplementary Information for:
Surface Polarization Profile of Ferroelectric Thin Films Probed by X-Ray Standing Waves and Photoelectron Spectroscopy

Le Phuong Hoang[1,2,3], Irena Spasojevic[4], Tien-Lin Lee[5], David Pesquera[6], Kai Rossnagel[3,7], Jörg Zegenhagen[5], Gustau Catalan[6,8], Ivan A. Vartanyants[9], Andreas Scherz[1], and Giuseppe Mercurio[1*]

[1]*European XFEL, 22869 Schenefeld, Germany*

[2]*Max Planck Institute for the Structure and Dynamics of Matter, 22761 Hamburg, Germany*

[3]*Institute of Experimental and Applied Physics, Kiel University, 24098 Kiel, Germany*

[4]*Department de Física, Universitat Autònoma de Barcelona, 08193 Bellaterra, Spain*

[5]*Diamond Light Source Ltd., Didcot, OX110DE Oxfordshire, United Kingdom*

[6]*Catalan Institute of Nanoscience and Nanotechnology (ICN2), CSIC and BIST, Campus UAB, Bellaterra, 08193 Barcelona, Spain*

[7]*Ruprecht Haensel Laboratory, Deutsches Elektronen-Synchrotron DESY, 22607 Hamburg, Germany*

[8]*Institucio Catalana de Recerca i Estudis Avançats (ICREA), 08010 Barcelona, Catalonia and*

[9]*Deutsches Elektronen-Synchrotron DESY, 22607 Hamburg, Germany*

[*] Corresponding author; giuseppe.mercurio@xfel.eu

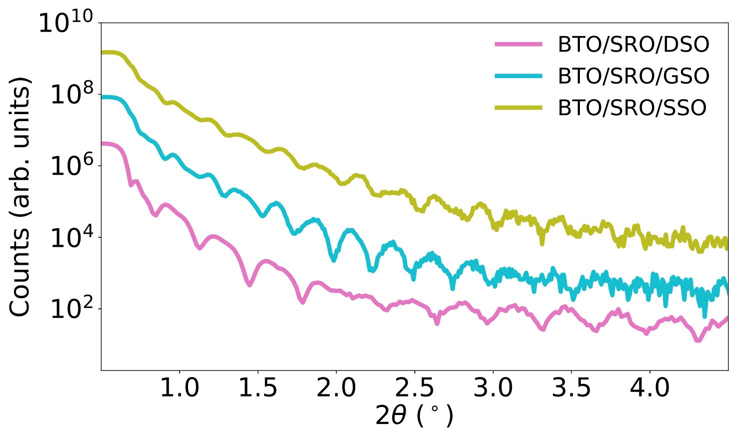

Supplementary Figure 1. Grazing X-ray reflectivity data of as-grown samples used to determine the thickness of the BTO and SRO thin films (Table 1 of the main text).

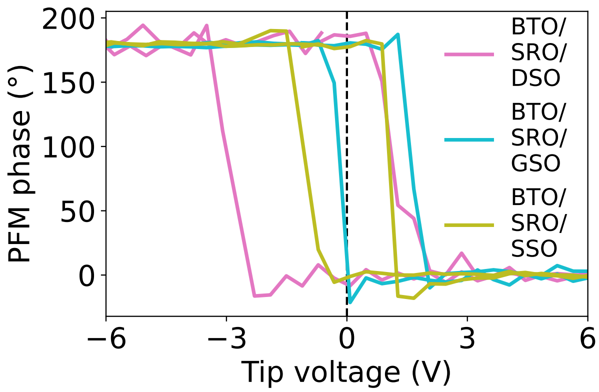

Supplementary Figure 2. Hysteresis loops obtained by switching spectroscopy PFM (SS-PFM). In the BTO/SRO/DSO sample, we measure a coercive voltage of $V_c = 1.1\,\mathrm{V}$ with a negative bias $V_b = -0.9\,\mathrm{V}$. In contrast, the BTO/SRO/GSO sample shows $V_c = 1.6\,\mathrm{V}$ with a positive bias $V_b = 0.7\,\mathrm{V}$. Finally, the BTO/SRO/SSO sample has an unbiased hysteresis loop with a coercive voltage $V_c = 1.1\,\mathrm{V}$.

Supplementary Table 1. BTO and SRO Debye-Waller factors e^{-W_i} of sublayers L_i $(i = 0, ..., 4)$ resulting from the fits of (001) Bragg reflection data in the three samples under study (Figure 3 of the main text).

Layer	sublayer	fit parameters	BTO/SRO/DSO	BTO/SRO/GSO	BTO/SRO/SSO
	L_0	e^{-W_0}	1	0.9	1
	L_1	e^{-W_1}	1	1	1
BTO	L_2	e^{-W_2}	1	1	0.9
	L_3	e^{-W_3}	1	1	1
	L_4	e^{-W_4}	1	0.1	0.5
	L_0	e^{-W_0}	0.5	0.7	0.9
	L_1	e^{-W_1}	1	0.7	1
SRO	L_2	e^{-W_2}	1	0.9	0.6
	L_3	e^{-W_3}	0.01	1	0.5
	L_4	e^{-W_4}	0	0	0.7

Supplementary Table 2. BTO and SRO interface strain ϵ_{int}, penetration depth of strain δ (nm), and rate of strain change δ_{lin} resulting from the fits of (001) Bragg reflection data in the three samples under study (Figure 3 of the main text).

Layer	fit parameters	BTO/SRO/DSO	BTO/SRO/GSO	BTO/SRO/SSO
BTO	ϵ_{int}	0.02	0.03	0.01
	δ	1.5×10^{-4} (δ_{lin})	139	237
SRO	ϵ_{int}	-0.01	-0.05	-0.04
	δ	124	57	78

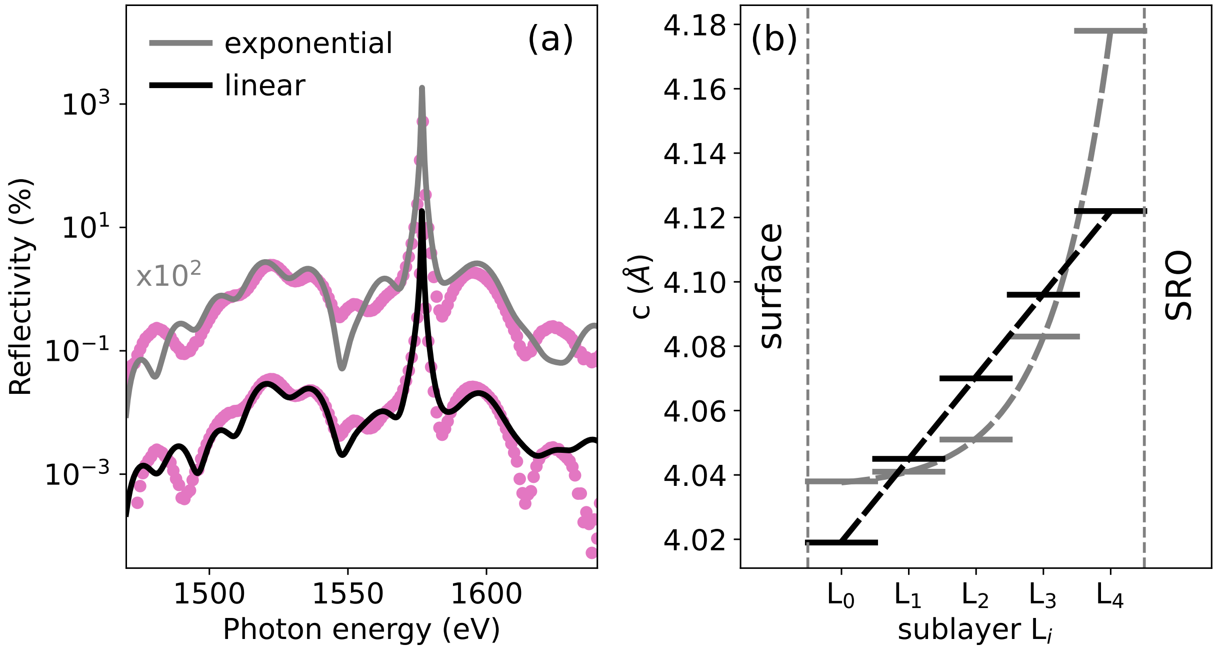

Supplementary Figure 3. (a) (001) Bragg reflectivity $R_0(E_\nu)$ of the BTO/SRO/DSO sample (pink points) and fit curves based on the exponential (gray) and linear (black) distribution of out-of-plane lattice parameters c displayed in panel b. The linear distribution of c parameters provides a fit of $R_0(E_\nu)$ near the BTO peak slightly better that the exponential c distribution, with residuals smaller than 5×10^{-3} %. The exponential c distribution is characterized by $\epsilon_{\text{int}} = 0.06$ and $\delta = 36$ nm. The XSW results of the two models for γ_1, γ_2, γ_3 are: $\Delta z_\gamma^{\text{Ti}} = 20$ pm, 16 pm, 8 pm (exponential model), and $\Delta z_\gamma^{\text{Ti}} = 16$ pm, 12 pm, 8 pm (linear model). Both models provide the same trend and differ in the absolute value only by 4 pm for γ_1 and γ_2. This corroborates the XSW results obtained and confirms their robustness against small changes in the structural model.

Supplementary Note 1. SRO structural properties

The in-plane strain applied by a substrate to the BTO thin film is calculated as $\epsilon^a_{\mathrm{SRO}} = (a_{\mathrm{SRO}} - a_{\mathrm{b,SRO}})/a_{\mathrm{b,SRO}}$, by comparing the measured in-plane lattice parameter of the thin film a_{SRO} (see Results of the main text) with the respective bulk value of the pseudocubic cell with parameter $a_{\mathrm{b,SRO}} \approx c_{\mathrm{b,SRO}} = 3.923\,\text{Å}$ [1]. As a result, the DSO, GSO, and SSO substrates impose an in-plane tensile strain on the SRO films of 0.51%, 1.12%, and 1.38%, respectively. This leads to a decreasing average out-of-plane lattice parameter in SRO following the same substrate order: $\bar{c}_{\mathrm{SRO}} = 3.885(19)\,\text{Å},\ 3.876(24)\,\text{Å},\ 3.866(23)\,\text{Å}$. These measured $\bar{c}$ parameters correspond to an average out-of-plane compressive strain $\bar{\epsilon}^c_{\mathrm{SRO}} = (\bar{c}_{\mathrm{SRO}} - c_{b,\mathrm{SRO}})/c_{b,\mathrm{SRO}}$ of −0.96%, −1.20%, and −1.45%, respectively. Figure **Supplementary Figure 4** shows the exponential distribution of SRO out-plane lattice parameters c_i (Equation 4 of the main text) in different sublayers L_i, resulting from the (001) Bragg reflections fits shown in Figure 3a of the main text.

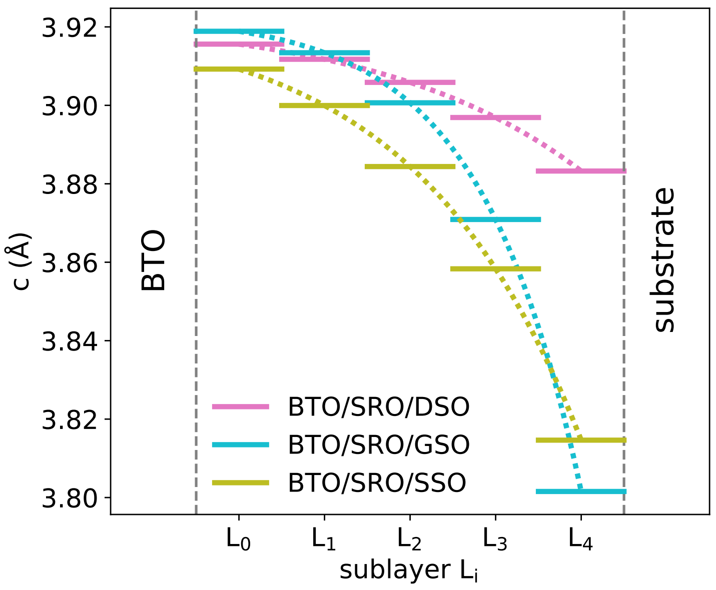

Supplementary Figure 4. SRO out-of-plane lattice parameters c_i (solid lines) in sublayers L_i and $c(z)$ (dotted lines) according to Equation (4) of the main text.

Supplementary Note 2. Ti 2p, Ba 4d and C 1s XPS

Supplementary Figure 5 shows typical PE spectra Ti 2p, Ba 4d, and C 1s core levels of the BTO/SRO/GSO and BTO/SRO/SSO samples. Ti and Ba PE spectra show similar features as the spectra in Figure 4 of the main text. The larger noise of PE spectra in **Supplementary Figure 5**d-e results from the lower PE intensity due to the larger amount of C and O species on the BTO/SRO/SSO surface. In fact, the corresponding C 1s spectrum has an area approximately three times larger than of the one of the BTO/SRO/GSO sample

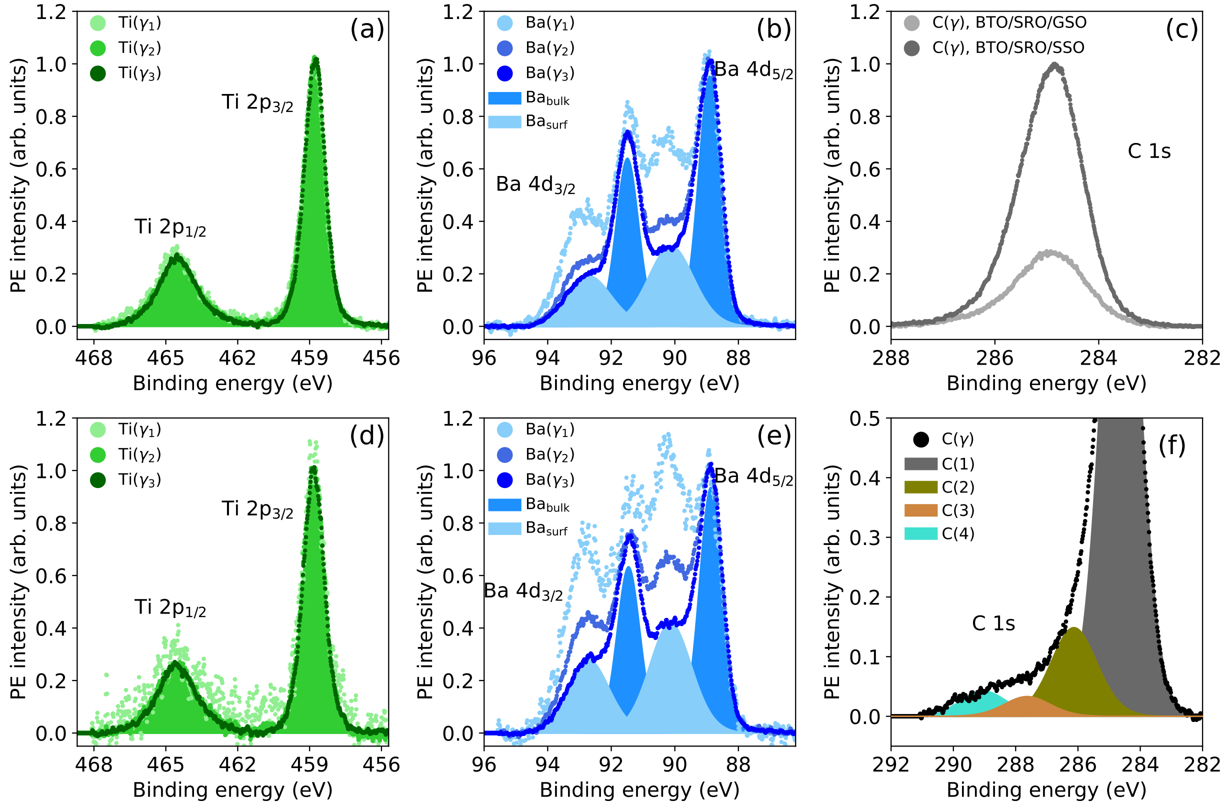

Supplementary Figure 5. Ti 2p and Ba 4d PE spectra of the BTO/SRO/GSO sample (a, b) and the BTO/SRO/SSO sample (d, e), respectively. Ti 2p and Ba 4d spectra are shown at the exit angle ranges γ_1, γ_2, and γ_3. Panel c shows the sum of C 1s spectra over all measured exit angle ranges of the BTO/SRO/GSO sample and the BTO/SRO/SSO sample. Note that both C 1s spectra are normalized to the peak intensity of C 1s spectrum the BTO/SRO/SSO sample to underscore the different content of carbon species in the two samples. Panel f shows a typical C 1s spectrum fitted with four components (see text).

(**Supplementary Figure 5**c). In particular, a typical C 1s spectrum, displayed in **Supplementary Figure 5**f, shows four components. Component C(1) at 284.6 eV is assigned to adventitious carbon, resulting from hydrocarbons adsorbed on the sample surface, upon exposure to ambient environment. Components C(2) and C(3), with BE shift of 1.5 eV and 3 eV, refer to C atoms bound by a single (C-O) or double (C=O) bond to an O atom [2, 3]. Component C(4), with BE shift of 4.4 eV, can be related to C atoms in carboxyl or ester groups [C-(C=O)-O], or in carbonate compounds [2, 3].

Supplementary Note 3. XPS fit results

PE spectra were measured using a fixed mode of the electron analyzer with a pass energy of 200 eV [100 eV] for Ba 4d, Ti 2p, C 1s [O 1s] core-level emission lines. In general, all PE spectra in this work were fitted using the software CasaXPS with Shirley background subtraction and a combination of Gaussian/Lorentzian functions with the best fit provided by the ratio 70/30 (for Ba 4d, O 1s, C 1s) and 40/60 (for Ti 2p). A summary of the resulting BE shifts and FWHM of each component is reported in **Supplementary Table 3**. In O 1s spectra, component P(5) can be associated with molecular physisorbed water or C-O bonds [2, 3], and has a minor contribution to the total spectral area (smaller than 3%).

Supplementary Table 3. FWHM of each fit component and BE shift of components P(m) ($m = 2, 3, 4, 5$) from component P(1) in Ba 4d, Ti 2p, O 1s and C 1s PE spectra.

	Ba 4d		Ti 2p		O 1s		C 1s	
	BE shift (eV)	FWHM (eV)	BE shift (eV)	FWHM (eV)	BE shift (eV)	FWHM (eV)	BE shift (eV)	FWHM (eV)
P(1)	–	0.83	–	1.02	–	1.14	–	1.41
P(2)	2.58	0.83	5.72	2.04	1	1.36	1.5	1.75
P(3)	1.21	1.65			2	1.32	3	1.75
P(4)	3.80	1.65			2.7	1.54	4.4	1.75
P(5)					3.85	1.45		

Supplementary Note 4. $\mathrm{Ba_{surf}}$**-O correlation**

Supplementary Figure 6 shows a correlation between the PE area of $\mathrm{Ba_{surf}}$ component and the sum of PE areas of O(2) and O(4) components. Component O(3) has a weaker correlation with $\mathrm{Ba_{surf}}$, as demonstrated by the fact that the sample BTO/SRO/DSO has a $\mathrm{Ba_{surf}}$ component (Figure 4b of the main text), despite the absence of the O(3) component in the respective O 1s PE spectrum (Figure 5a of the main text).

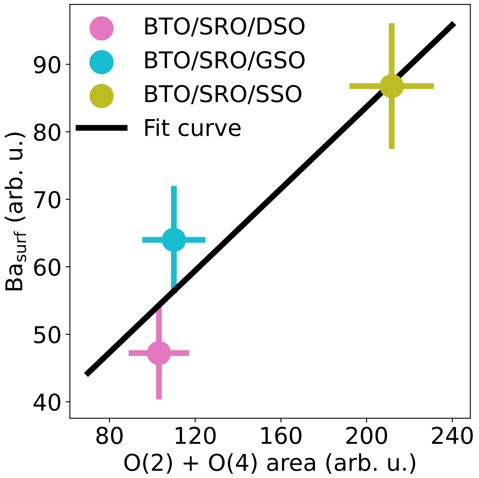

Supplementary Figure 6. Plot of PE area of $\mathrm{Ba_{surf}}$ component as a function of the sum of PE areas of O(2) and O(4) components together with the linear fit curve.

Supplementary Note 5.   Photoelectron yield normalization

The PE yield undergoes two normalization steps: (i) by the incident X-ray intensity, and (ii) by the photoionization cross section. First, the X-ray intensity I_0 is measured as the drain current from the last mirror before the sample. The incident X-ray intensity I_0 decreases by approximately 10% in the energy range from 1400 to 1700 eV (**Supplementary Figure 7**b). This is due to the decreasing monochromator grating efficiency. Furthermore, the I_0 sawtooth profile in the region from 1480 to 1620 eV results from the top-up electron injection at the Diamond Light Source. PE yield data normalized by I_0 are shown in **Supplementary**

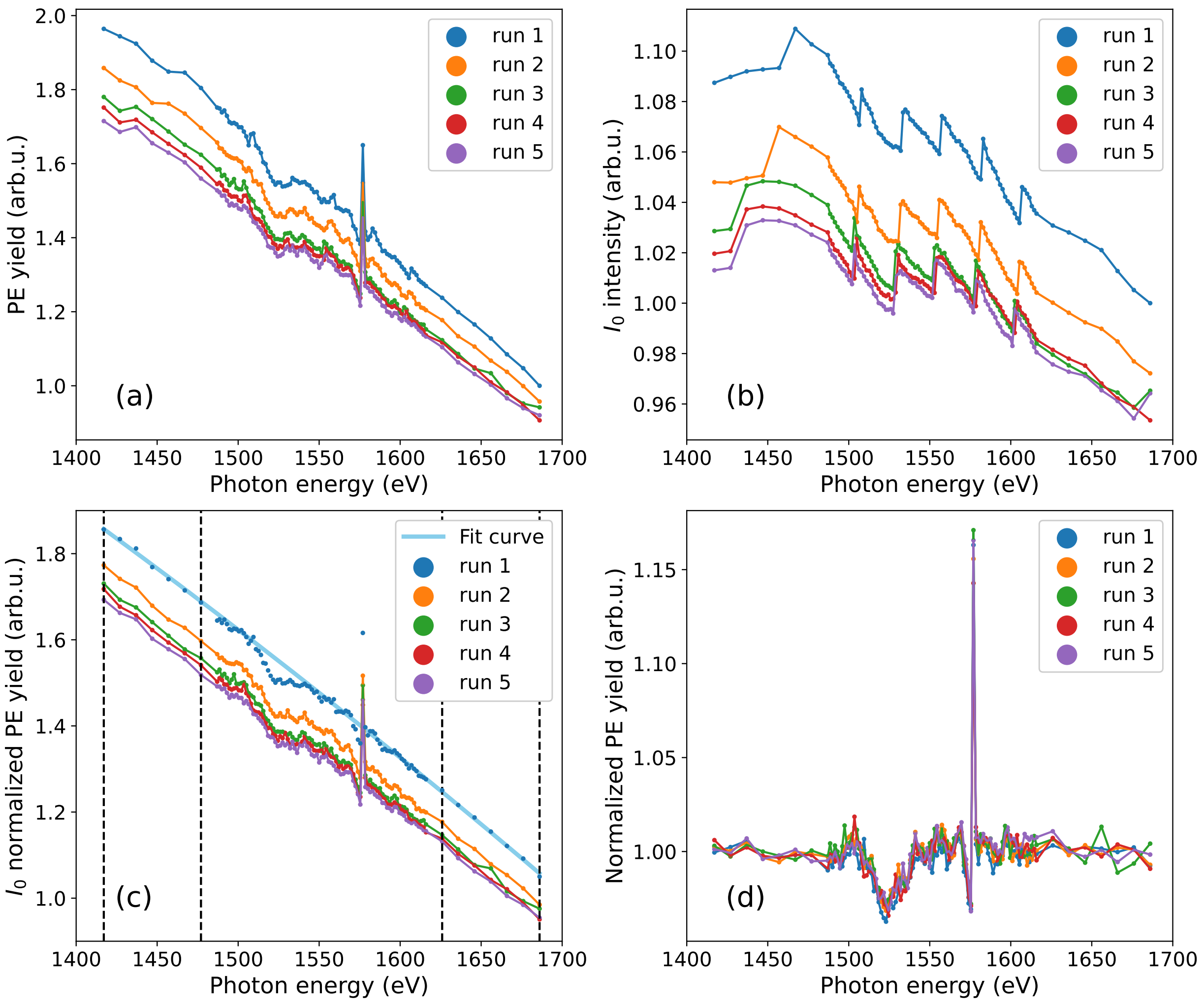

Supplementary Figure 7. (a) Ba 4d PE yield raw data of the BTO/SRO/DSO sample measured in 5 consecutive photon energy scan (run). (b) Incident X-ray beam intensity I_0. (c) PE yield raw data normalized by I_0. (d) PE yield raw data normalized by I_0 and photoionization cross section.

Figure 7c. Here, the decrease in PE yield by a factor ≈ 1.8 over the whole photon energy range follows from the varying photoionization cross section [4, 5]. The second normalization step consists in dividing PE yield data by a second order polynomial resulting from the fit of 7 points at each end of a yield curve in **Supplementary Figure 7**c, where no XSW effect is observed. An example of fitting curve is shown in **Supplementary Figure 7**c (blue solid line), while the normalized PE yield curves are reported in **Supplementary Figure 7**d. Finally, at each photon energy E_ν, average and standard deviation of PE yield data (measured under the same conditions) are calculated to determine the PE yield values and error bars reported in Figure 6 of the main text.

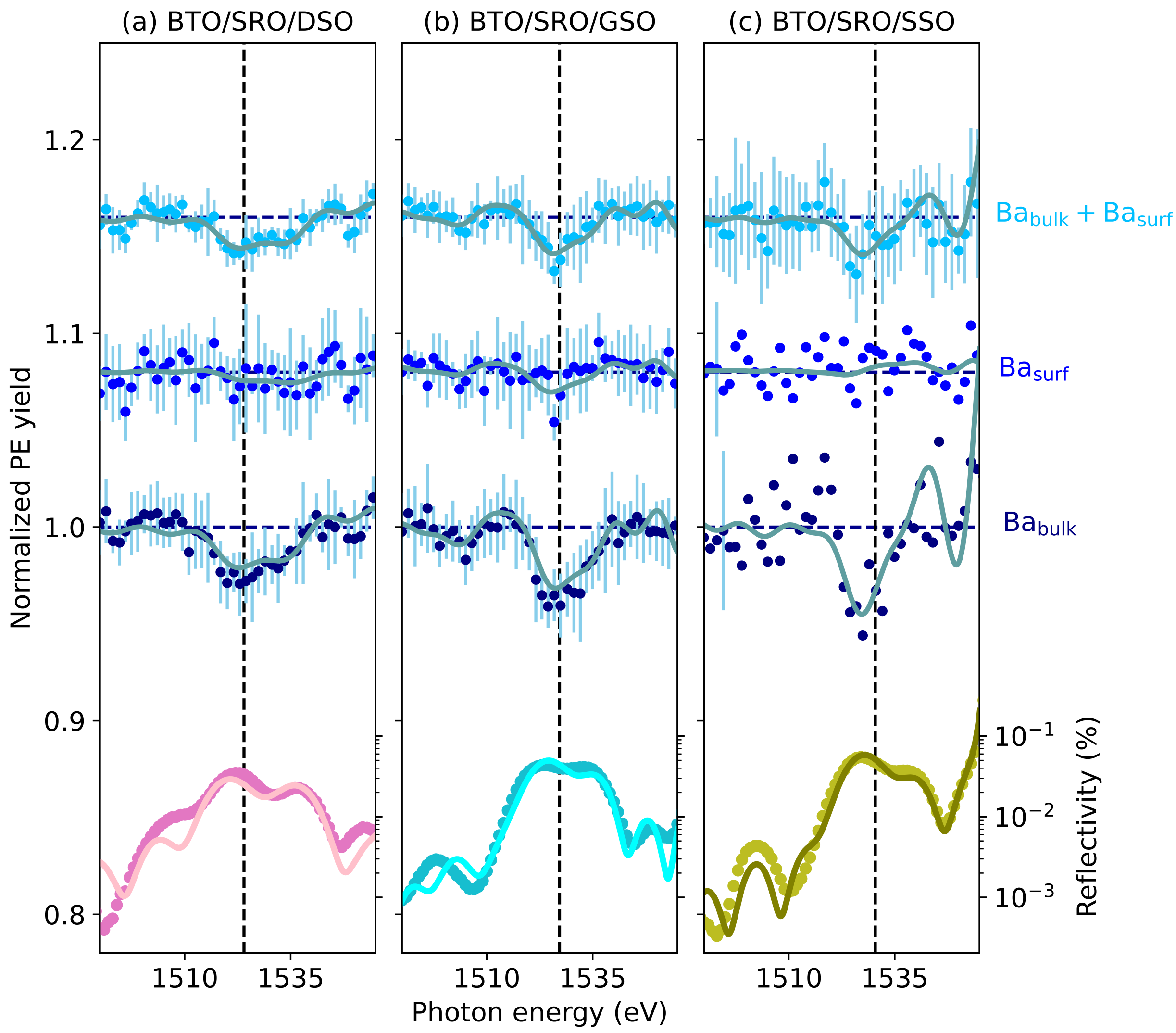

Supplementary Figure 8. Ba PE yield data of components Ba_{surf}, Ba_{bulk} and Ba_{bulk} + Ba_{surf}, measured at the exit angle range γ_1 on BTO/SRO/DSO (a), BTO/SRO/GSO (b), and BTO/SRO/SSO (c), and corresponding fit curves (solid lines). Reflectivity $R_0(E\nu)$ data and fit curves around the (001) BTO Bragg energies E_B = 1524 eV (a), 1527.2 eV (b), 1530.4 eV (c) (marked by vertical dashed lines). For clarity, $\kappa_{\gamma_1}^{\mathrm{Ba_{surf}}}(E_\nu)$ and $\kappa_{\gamma_1}^{\mathrm{Ba_{bulk}}}(E_\nu)$ of BTO/SRO/SSO are shown with only one error bar, which corresponds to the average error bar of all $\kappa_{\gamma_1}^{\mathrm{Ba_x}}(E_\nu)$ data points, with x = bulk or surf. All PE yield curves are normalized (see Supplementary Note 5) and, for clarity, the curves above Ba(γ_3) are vertically shifted by 0.08 from the one below.

[1] C. L. Jia, J. R. Contreras, U. Poppe, H. Kohlstedt, R. Waser, and K. Urban, Lattice strain and lattice expansion of the $SrRuO_3$ layers in $SrRuO_3/PbZr_{0.52}Ti_{0.48}O_3/SrRuO_3$ multilayer thin films, J. Appl. Phys. **92**, 101 (2002), https://doi.org/10.1063/1.1483369.

[2] J. Landoulsi, M. J. Genet, S. Fleith, Y. Touré, I. Liascukiene, C. Méthivier, and P. G. Rouxhet, Organic adlayer on inorganic materials: XPS analysis selectivity to cope with adventitious contamination, Appl. Surf. Sci. **383**, 71 (2016), https://www.sciencedirect.com/science/article/pii/S0169433216309357.

[3] I. Spasojevic, G. Sauthier, J. M. Caicedo, A. Verdaguer, and N. Domingo, Oxidation processes at the surface of $BaTiO_3$ thin films under environmental conditions, Appl. Surf. Sci. **565**, 150288 (2021), https://www.sciencedirect.com/science/article/pii/S0169433221013635.

[4] M. Trzhaskovskaya, V. Nefedov, and V. Yarzhemsky, Photoelectron angular distribution parameters for elements Z=1 to Z=54 in the photoelectron energy range 100–5000 eV, At. Data Nucl. Data Tables **77**, 97 (2001), https://www.sciencedirect.com/science/article/pii/S0092640X00908490.

[5] M. Trzhaskovskaya, V. Nefedov, and V. Yarzhemsky, Photoelectron angular distribution parameters for elements Z=55 to Z=100 in the photoelectron energy range 100–5000 eV, At. Data Nucl. Data Tables **82**, 257 (2002), https://www.sciencedirect.com/science/article/pii/S0092640X02908867.
